# CMYC-initiated HNF1A-AS1 overexpression maintains the stemness of gastric cancer cells

**DOI:** 10.1038/s41419-024-06673-y

**Published:** 2024-04-23

**Authors:** Ruinan Zhao, Xiangyu Guo, Guohao Zhang, Sen Liu, Ranran Ma, Mengqi Wang, Shiming Chen, Wenjie Zhu, Yuan Liu, Peng Gao, Haiting Liu

**Affiliations:** 1 Department of Pathology, Qilu Hospital and School of Basic Medical Sciences, Shandong University, Jinan, China; 2 Department of Obstetrics and Gynecology, Qilu Hospital, Shandong University, Jinan, China

**Keywords:** Gastric cancer, Cancer stem cells

## Abstract

Cancer stem cells (CSCs) are believed to be responsible for cancer metastasis and recurrence due to their self-renewal ability and resistance to treatment. However, the mechanisms that regulate the stemness of CSCs remain poorly understood. Recently, evidence has emerged suggesting that long non-coding RNAs (lncRNAs) play a crucial role in regulating cancer cell function in different types of malignancies, including gastric cancer (GC). However, the specific means by which lncRNAs regulate the function of gastric cancer stem cells (GCSCs) are yet to be fully understood. In this study, we investigated a lncRNA known as HNF1A-AS1, which is highly expressed in GCSC s and serves as a critical regulator of GCSC stemness and tumorigenesis. Our experiments, both in vitro and in vivo, demonstrated that HNF1A-AS1 maintained the stemness of GC cells. Further analysis revealed that HNF1A-AS1, transcriptionally activated by CMYC, functioned as a competing endogenous RNA by binding to miR-150-5p to upregulate β-catenin expression. This in turn facilitated the entry of β-catenin into the nucleus to activate the Wnt/β-catenin pathway and promote CMYC expression, thereby forming a positive feedback loop that sustained the stemness of GCSCs. We also found that blocking the Wnt/β-catenin pathway effectively inhibited the function of HNF1A-AS1, ultimately resulting in the inhibition of GCSC stemness. Taken together, our results demonstrated that HNF1A-AS1 is a regulator of the stemness of GCSCs and could serve as a potential marker for targeted GC therapy.

## Introduction

According to the latest global cancer study released by the World Health Organization in 2020, gastric cancer (GC) has become the third most common cancer in China and the third leading cause of death worldwide [1]. Unfortunately, advanced GC often results in death due to distant metastases or recurrence [2]. Consequently, there is an urgent need for effective measures to prevent GC metastasis and recurrence.

Recent studies have revealed that long non-coding RNAs (lncRNAs) play an important role in various aspects of tumors, in addition to the abnormal expression of genes encoding proteins [3, 4]. However, the roles of lncRNAs in cancer stem cells (CSCs) are not yet clear.

CSCs are a subset of cells with distinct roles and properties that cause cancer genesis, therapeutic tolerance, aggressive tumor growth, metastasis, and recurrence [5–7]. Targeting CSCs is effective in various cancers. Targeting CD276 to eliminate CSCs suppressed head and neck squamous cell carcinoma development and metastasis [8]. In breast cancer, suppressing the niche formation of CSCs reduced their enrichment in the tumor and reversed drug resistance [9]. In malignant peripheral nerve sheath tumors, targeting CSCs reduced tumor recurrence after chemotherapy [10]. Thus, understanding the mechanisms of stemness maintenance in CSCs is critical to developing effective cancer treatment strategies.

In our previous study, we identified associations between the progression of GC and several lncRNAs, including HNF1A-AS1, WT1AS, TP53TG1, and HTMGC [11]. In the present study, HNF1A-AS1 was specifically found to be involved in the stemness of GC cells. Building on this, we investigated the role of HNF1A-AS1 in GC initiation. HNF1A-AS1 is the antisense sequence of hepatocyte nuclear factor-1α (HNF-1α). HNF-1α is a transcription factor that is mainly expressed in the liver and other organs and has an important role in the development of liver tumors [12]. This study demonstrated that HNF1A-AS1 was increased in gastric cancer stem cells (GCSCs) and promoted stemness. We found that HNF1A-AS1 competed with miR-150-5p as a competing endogenous RNA (ceRNA) to upregulate β-catenin and that CMYC upregulated β-catenin transcriptionally. Finally, HNF1A-AS1 regulated GCSC stemness via miR-150-5p, β-catenin, and CMYC in a positive feedback loop.

## Materials and methods

### Cell lines, cell culture and transfection

Human GC cell lines MKN-45 and BGC-823 were obtained from the Chinese Academy of Sciences (Shanghai, China). Cells were grown in RPMI-1640 media (HyClone) with 10% FBS (Gibco). These cells were tested for mycoplasma once a month using the MycoBlue Mycoplasma Detector (Vazyme). Cells were seeded in different well plates according to the experimental needs, and transfection was performed after 12 h when the cells were in logarithmic growth phase. TurboFect (Thermo Fisher Scientific) transfected plasmids into cells in this study. Cells were collected after 48H to extract RNA for quantitative real-time polymerase chain reaction (qRT-PCR) to determine the transfection efficiency, using the corresponding vector plasmid (PCDNA3.1) as a control group.

### Cell lentiviral transduction

The 96-well plates were seeded with 5000 cells per well and transfected at 12 h. LV-HNF1A-AS1, as well as the blank control LV-NC, were transduced into MKN-45 and BGC-823 cells, respectively, following the lentiviral instructions (GenePharma). After 24 h, the medium was replaced with normal medium, and after 72 h, the cells were observed under fluorescence microscopy for green fluorescence. The culture of lentivirus-infected cells was expanded. LV-NC served as the control group, and cells were collected every seven days for RNA extraction to detect transduction efficiency using qRT-PCR analysis.

### RNA extraction and qRT-PCR

Total RNA was extracted from tissues and cultured cells using TRIzol® (Invitrogen), following the manufacturer’s instructions. Reverse transcription of approximately 1 μg of total RNA was performed using a reverse transcriptase cDNA synthesis kit (Toyobo), and qRT-PCR was conducted using a SYBR Green PCR kit (Roche). The 2^−ΔCt^ method was used to comparatively quantify gene expression, with GAPDH serving as the endogenous control.

### Aldehyde dehydrogenase activity assay

Aldehyde dehydrogenase (ALDH) activity was detected using an ALDEFLUOR kit (Stemcell) in accordance with the manufacturer’s instructions. Briefly, a single-cell suspension was prepared by adding ALDEFLOUR Buffer and 5 μl of Active ALDEFLOUR Reagent, depending on the number of cells. Half of the mixed system was then transferred to another centrifuge tube and 5 μl of diethylaminobenzaldehyde (DEAB), an inhibitor of ALDH, was added as a negative control. The system was incubated at 37 °C for 45 min, the supernatant was discarded by centrifugation, and the cells were resuspended by adding 500 μl of buffer and analyzed by flow cytometry.

### Sphere formation assay

Cancer cells were cultured at a density of 1000 cells per milliliter in ultra-low adhesion plates (Corning) in serum-free RPMI-1640 medium (HyClone) supplemented with B27 (1:50, Invitrogen), 20 ng/mL EGF (BD Biosciences), and 20 ng/mL FGF (BD Biosciences). Microspheres with a diameter greater than 75 μm were counted after culturing for 10 days. Next, we harvested these microspheres, with a portion used for subsequent experiments, while the remainder was enzymatically digested with trypsin into individual cells and counted. One thousand cells were then selected and continued to be cultured under the aforementioned conditions. This process was repeated three times, with the microspheres obtained from each repetition labeled sequentially as “1st”, “2nd”, and “3rd”. Each analysis included six replicate wells, and at least three independent experiments were conducted.

### Colony formation

Five hundred cells per well were planted in six-well plates. After 2 weeks, monoclonal cells were generated, fixed, dyed with crystal violet, and viewed under a microscope. Next, the experimental and control groups’ monoclonal cell numbers were calculated.

### CCK8 assay

Cells were seeded in 96-well plates with 4000 cells per well and 5 replicate wells were seeded for each set of experiments. Before the assay, 10 μl CCK8 assay solution (TargetMol) was added to each well and the cells were incubated for 2 h in the cell culture incubator. Absorbance values at 450 nm were read with an enzyme meter and cell activity was calculated.

### RNA binding protein immunoprecipitation (RIP) assay

The RIP assay followed Millipore’s Magna RIP RNA Binding Protein Immunoprecipitation Kit instructions. Cells were lysed in protease and RNase-inhibited lysis buffer. Anti-Ago2 and IgG antibodies immunoprecipitated lysates. qRT-PCR measured recoverable RNAs.

### Dual-luciferase reporter assay

Following the manufacturer’s instructions, a dual-luciferase reporter assay (Promega) was used to detect the firefly luciferase and renilla luciferase signals. Briefly, MKN-45 and BGC-823 cells were seeded overnight in 24-well plates and co-transfected with Lipofectamine 2000 (Invitrogen) using 0.5 μg of the overexpression vector, 0.5 μg of the HNF1A-AS1 promoter-luciferase reporter plasmid, and 0.01 μg of pRL-TK plasmid. Internal control was implemented using PRL-TK. After approximately 48 h of transfection, the luciferase activity of the cells was determined using a Dual-Luciferase Reporter Assay System (Promega) in accordance with the manufacturer’s instructions. Relative fluorescence values were expressed as firefly fluorescence values over renilla fluorescence values.

### Western blotting

The cells’ proteins were extracted with RIPA buffer, separated on SDS-polyacrylamide gels, and then transferred to PVDF membranes. For western blotting, the following antibodies were utilized: anti-OCT4 (Abcam, ab181557), anti-SOX2 (Abcam, ab92494), anti-Nanog (Proteintech, 14295-1-AP), anti-β-catenin (Abcam, ab32572), and anti-CMYC (Abcam, ab32072). A peroxidase-conjugated secondary antibody was used, and the antigen-antibody reaction was visualized using an enhanced chemiluminescence assay.

### Separate and extract cytoplasmic and nuclear proteins

Experiments were performed using the Nuclear and Cytoplasmic Protein Extraction kit (Beyotime). The cytoplasmic proteins were isolated first and the nuclear proteins were extracted in accordance with the instructions in the manual. For the western blotting assay, anti-GAPDH (Proteintech, 60004-1-Ig) was used as an internal reference for cytoplasmic proteins and anti-LaminB (Proteintech, 12987-1-AP) was used as an internal reference for nuclear proteins.

### Tumor xenograft model

BALB/c athymic nude male mice (4 weeks old) were obtained from Weitong Lihua Biotechnology (Beijing, China) and were used for subcutaneous injection of LV-HNF1A-AS1 and LV-NC-MKN-45-transfected cells into the flank region. The mice were randomly divided into 6 groups. LV-HNF1A-AS1 or LV-NC cells were injected subcutaneously into 4-week-old male nude mice (8 per group) at three different doses (2 × 10^5^, 2 × 10^4^, and 2 × 10^3^). After 6 weeks, the tumors were collected and RNA and proteins were extracted. All animal procedures complied with the Guide for the Care and Use of Laboratory Animals (2015 reprint) and were endorsed by the institutional ethical guidelines of Shandong University of Medicine.

### Clinical specimens

A total of 26 cases of fresh GC tissues were collected from Qilu Hospital of Shandong University (Shandong Province, China) and Shandong Provincial Hospital (Shandong Province, China) during 2012–2014. This study was approved by the Institute’s Research Ethics Committee of Shandong University and conducted in accordance with the ethical guidelines of the World Medical Association Declaration of Helsinki. Informed consent was written by all patients prior to this study.

### Immunohistochemsitry (IHC)

Formalin-fixed and paraffin-embedded stomach cancer samples were immunohistochemically examined. CMYC (Santa cruz, sc-40) and β-catenin (Santa cruz, sc-7963) antibodies were used to perform an overnight antigen-antibody reaction at 4 °C on deparaffinized tissues. Positive cytoplasm staining. Two pathologists independently graded cytoplasm staining from 0 (negative) to 3 (strong). Q = I1×P1 + I2×P2 + I3×P3 (I: intensity, P: percentage) was the histoscore (Q).

### TCGA database analysis

The TCGA database comprises global gene expression profiles of many cancers and corresponding non-tumor tissues, as well as genomic sequencing data from over 20 human malignancies. RNA-seq data with clinical information from the GDC TCGA Gastric Cancer cohort (https://portal.gdc.cancer.gov/) was scanned and extracted.

### Chromatin immunoprecipitation

According to manufacturer instructions, Magna ChIP kit (Sigma) was used for ChIP tests. Cells in 10 cm dishes were cross-linked with 1% formaldehyde for 10 min. To quench the fixation reaction, 10 × glycine buffer was added to the cell suspension at room temperature for 10 min. Cell pellets were resuspended in Protease Inhibitor Cocktail II lysis buffer after washing with PBS. Lysed cells were sonicated at 100 W, 30% power for 3 runs, then iced for 2 min for 8 runs. The lysate was immunoprecipitated with 5 μg of antibody. qRT-PCR templates were purified DNA fragments.

### Statistical analysis

Statistical analyses were conducted using GraphPad Prism 8.0 (San Diego, Inc., CA, USA). One-way ANOVA, two-way ANOVA or Student’s *t*-test was employed based on sample characteristics. For tumor-initiating cell (TIC) frequency, ELDA software was used with Pearson’s chi-squared two-tailed test. Spearman’s correlation coefficient was applied to analyze the correlation between HNF1A-AS1 and CMYC, HNF1A-AS1 and β-catenin relative expression in clinical samples. The data are expressed as mean ± SD. Each experiment was repeated at least three times unless otherwise noted. *P* values less than 0.05 were considered statistically significant.

## Results

### HNF1A-AS1 is associated with the stemness of GCSCs

**Fig. 1:**
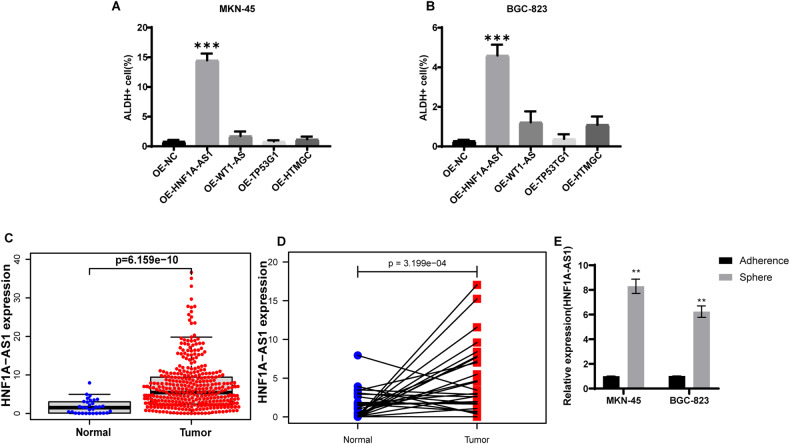
HNF1A-AS1 is upregulated in GCSCs and correlates with stemness. **A**, **B** Quantification plots showed the proportion of ALDH+ cells in MKN-45 and BGC-823 after overexpression of HNF1A-AS1, WT1AS, TP53TG1, HTMGC and PCDNA3.1, respectively (*n* = 3). **C** Expression of HNF1A-AS1 in a dataset containing tumors and normal tissues from TCGA (Tumor number = 375 normal number = 32). **D** Expression of HNF1A-AS1 in paired carcinomas and surrounding normal tissues from the TCGA database (*n* = 27). **E** Relative quantification of mRNA expression of HNF1A-AS1 in spheres compared with adherent cells. Data are representative as the mean ± SD. One-way ANOVA with Tukey’s multiple-comparison test (**A**, **B**), two-tailed unpaired Student’s t-test (**C,** **E**), two-tailed paired Student’s *t*-test (**D**). *P < 0.05; **P < 0.01; ***P < 0.001.

To identify lncRNAs associated with the stemness of GC cells, we selected HNF1A-AS1, WT1AS, TP53TG1, and HTMGC, based on our previous research results [11]. A tumor marker for CSCs, ALDH was shown to be closely related to tumor malignancy and patient prognosis [13, 14]. Thus, we overexpressed HNF1A-AS1, WT1AS, TP53TG1, and HTMGC in two GC cell lines, MKN-45 and BGC-823, to evaluate whether these lncRNAs influenced GC cell stemness. Forty-eight hours after overexpression, flow cytometry was used to evaluate ALDH activity (Supplementary Fig. 1A, B). None of the lncRNAs affected GC cell stemness except for HNF1A-AS1, which increased ALDH activity in GC cells (Fig. 1A, B). Furthermore, analysis of The Cancer Genomic Atlas (TCGA) database revealed that HNF1A-AS1 expression was higher in GC tissue than in normal tissue samples (Fig. 1C). We analyzed the expression levels of HNF1A-AS1 in paired gastric cancer and surrounding normal tissue in TCGA database. The results showed that in the same sample, the expression level of HNF1A-AS1 was higher in gastric cancer tissue than in the surrounding normal tissue (Fig. 1D). We then proceeded to establish an in vitro proliferation model of GCSCs using a tumor microsphere formation assay. In this model, tumor cells with stemness can be grown in a sphere-forming suspension in serum-free medium in ultra-low adsorption dishes. The results of qRT-PCR showed an increased expression of HNF1A-AS1 in GC cells with stemness (Fig. 1E).

**Fig. 2:**
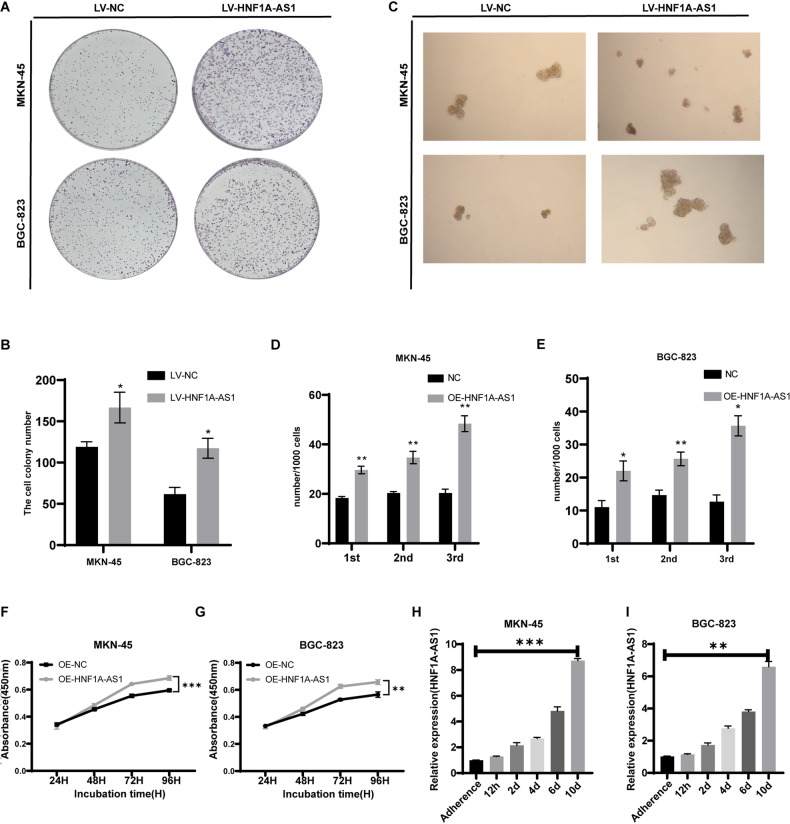
Overexpression of HNF1A-AS1 promotes self-renewal of GCSCs in vitro. **A** Colony formation assays were used to explore the cell colony formation ability of LV-HNF1A-AS1 and LV-NC-transfected cells. **B** The bar graph showed the number of gastric cancer cell clones formed (*n* = 3). **C** Representative images of GCSC spheres overexpressing LV-HNF1A-AS1. Original magnification, ×40. **D**, **E** The bar graph showed the number of spheres formed (*n* = 3). **F**, **G** CCK8 assay to investigate the effect of overexpression of HNF1A-AS1 on the proliferative capacity of GC cells (*n* = 3). **H**, **I** qRT-PCR analysis of the expression of HNF1A-AS1 in MKN-45 and BGC-823 cells after 12 h, 2, 4, 6 and 10 d of spheroid formation (*n* = 3). Data are representative as the mean ± SD. Two-tailed unpaired Student’s t-test (**B**, **D**, **E**), two-way ANOVA test (**F**, **G**), one-way ANOVA with Tukey’s multiple-comparison test (**H**, **I**). *P < 0.05; **P < 0.01; ***P < 0.001.

To study the role of HNF1A-AS1 in GC cells, we generated cells with stable overexpression of HNF1A-AS1. Monoclonal formation experiments demonstrated that the overexpression of HNF1A-AS1 caused a considerably larger number of cell clones than did the control group (Fig. 2A, B). Similarly, the number of microsphere formations in GC cells was increased in the HNF1A-AS1 overexpression group compared with the control group (Fig. 2C–E), suggesting that HNF1A-AS1 maintained the self-renewal of GC cells. Coinciding with our cell clone results, CCK8 assay results also showed that HNF1A-AS1 enhanced cell proliferation (Fig. 2F, G). To further investigate the relationship between HNF1A-AS1 expression and the stemness of GC cells, we collected cells at 12 h and 2, 4, 6, and 10 days using an expansion model of GCSCs. qRT-PCR analysis revealed that the expression of HNF1A-AS1 in sphere-forming GC cells gradually increased with time (Fig. 2H, I). These results indicate that HNF1A-AS1 is highly expressed in GCSCs and is involved in maintaining the stemness of GC cells.

### HNF1A-AS1 is essential for the initiation of gastric xenograft tumor in vivo

**Fig. 3:**
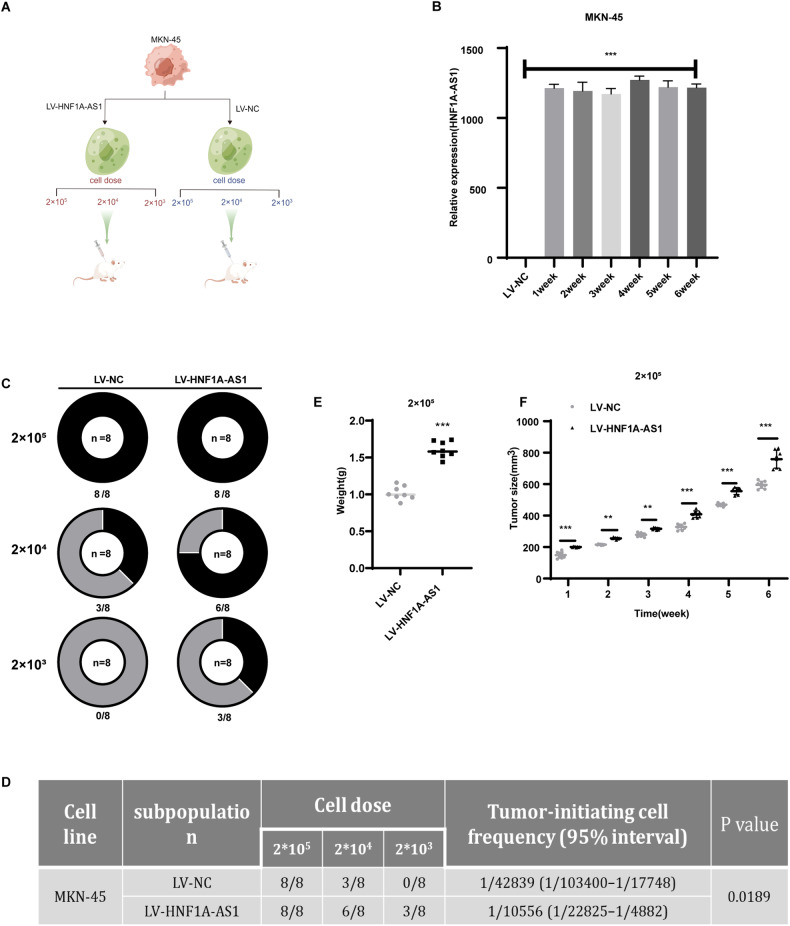
HNF1A-AS1 facilitates the tumor initiation ability of GC in vivo. **A** Mice subcutaneously were injected with GC cells by three doses, then the xenograft tumors were harvested. **B** Transduction efficiency and duration of LV-HNF1A-AS1 virus detected by qRT-PCR (*n* = 3). **C** Tumor formation in each group of mice after six weeks (*n* = 8). The black area portion represents the number of mice that formed tumors. **D** The frequency of TIC was calculated based on the positive tumor site in each group. TIC frequency was calculated by ELDA software. **E** At 6 weeks after the subcutaneous injection, tumor weight was measured (*n* = 8). **F** Tumor volume was measured weekly (*n* = 8). Data are representative as the mean ± SD. One-way ANOVA with Tukey’s multiple-comparison test (**B**), Pearson’s chi-squared two-tailed test (**D**), two-tailed unpaired Student’s *t*-test (**E**), two-way ANOVA test (**F**). *P < 0.05; **P < 0.01; ***P < 0.001.

CSCs play a crucial role in tumor initiation [15]. We aimed to investigate whether HNF1A-AS1 is involved in regulating the ability of GC cells to initiate tumors. To examine the role of HNF1A-AS1 in regulating the stemness of GC cells in vivo, we first transfected MKN-45 cells with LV-HNF1A-AS1 or LV-NC. Subsequently, we subcutaneously injected cells transfected with LV-HNF1A-AS1 or LV-NC into mice (*n* = 8 per group) at three different doses (2 × 10^5^, 2 × 10^4^, and 2 × 10^3^ cells) (Fig. 3A). We continued to culture the cells remaining after inoculation of the mice. Then we collected and extracted RNA from MKN-45 cells weekly after transfection with LV-HNF1A-AS1 to detect the expression level of HNF1A-AS1. The results showed that the LV-HNF1A-AS1-transfected MKN-45 cells stably overexpressed HNF1A-AS1 (Fig. 3B). After 6 weeks, we observed and recorded the formation of xenogeneic tumors in each group and collected the tumors. Our results indicated that LV-NC required at least 2 × 10^4^ cells to form tumors in nude mice, whereas 2 × 10^3^ cells from LV-HNF1A-AS1-transfected cells were sufficient to form tumors in nude mice (Fig. 3C). Additionally, GC cells with high expression of HNF1A-AS1 exhibited higher tumor initiation in nude mice (Fig. 3D). Furthermore, HNF1A-AS1 overexpression enhanced tumor size and weight in vivo (Fig. 3E, F). This suggests that HNF1A-AS1 plays a crucial role in maintaining the stemness of GCSCs and promoting the tumor initiation capability of GC cells in vivo.

### HNF1A-AS1 maintains the stemness of GCSCs through the Wnt/β-catenin pathway

**Fig. 4:**
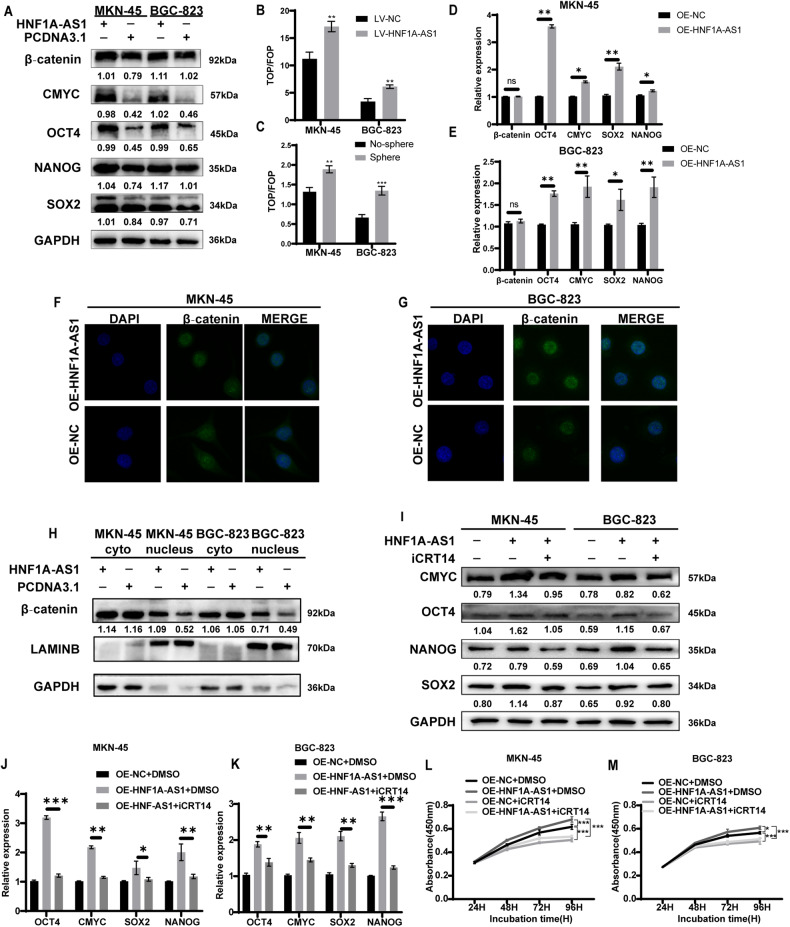
HNF1A-AS1 maintains stemness of GCSCs through Wnt/β-catenin pathway. **A** Western blotting assay was used to identify the effect of HNF1A-AS1 on cancer stemness markers. **B**, **C** TOP/FOP-flash reporter plasmid was transfected in GC cells, and luciferase activity was detected (*n* = 3). **D**, **E** qRT-PCR analysis of β-catenin, CMYC, OCT4, SOX2 and NANOG expression in MKN-45 and BGC-823 cells overexpressing HNF1A-AS1 or negative control (*n* = 3). **F**, **G** Immunofluorescence staining assay indicating the localization of β-catenin in GC cells. Confocal micrographs of β-catenin. Original magnification, ×630. **H** Western blotting assay was used to determine the effect of HNF1A-AS1 on the intracellular localization of β-catenin. **I**–**K** The expression of CMYC, OCT4, SOX2 and NANOG in MKN-45 and BGC-823 cells treated with iCRT14 and DMSO were detected by western blotting and qRT-PCR. **L**, **M** CCK8 assay to detect cell viability after addition of the Wnt/β-catenin pathway inhibitor iCRT14 (*n* = 3). Data are representative as the mean ± SD. Two-tailed unpaired Student’s *t*-test (**B**–**E**), one-way ANOVA with Tukey’s multiple-comparison test (**J**, **K**), two-way ANOVA test (**L**, **M**). *P < 0.05; **P < 0.01; ***P < 0.001.

Several signaling pathways, such as Wnt/β‐catenin, Notch, and Hedgehog, have been reported to regulate the stemness of GCSCs [16–19]. Interestingly, overexpression of HNF1A-AS1 led to increased β‐catenin protein levels and associated stemness markers (Fig. 4A). Further quantitative protein analysis revealed a significant increase in the levels of the Wnt/β-catenin pathway and downstream stemness-related proteins in GCs overexpressing HNF1A-AS1 (Supplementary Fig. 2A, B). The values of the TOP/FOP fluorescence system were enhanced in both gastric cancer cells overexpressing HNF1A-AS1 and in cancer cell spheres with stemness (Fig. 4B, C). This suggests that gastric cancer cells overexpressing HNF1A-AS1 activate the Wnt/β‐catenin pathway, similar to cancer cell spheres with stemness.

Next, we investigated the mRNA expression of Wnt/β-catenin pathway markers in GC cells. Our results showed that overexpression of HNF1A-AS1 elevated the levels of *CMYC*, *OCT4*, *SOX2*, and *NANOG*, but did not significantly affect *β-catenin* mRNA levels (Fig. 4D, E). β-Catenin accumulates in the cytoplasm and eventually translocates into the nucleus, further activating the Wnt/β-catenin signaling pathway. Based on the mechanism of the Wnt/β-catenin pathway, we hypothesized that HNF1A-AS1 facilitates the entry of β-catenin into the nucleus. Fluorescence immunolocalization experiments confirmed that overexpression of HNF1A-AS1 led to nuclear translocation of β-catenin (Fig. 4F, G). Moreover, HNF1A-AS1 transfection enhanced β-catenin protein expression in the nucleus (Fig. 4H and Supplementary Fig. 2C, D). To determine if the elevated expression of *CMYC*, *OCT4*, *SOX2*, and *NANOG* was mediated by the canonical Wnt/β-catenin pathway, we treated GC cells with the β-catenin/TCF inhibitor iCRT14 to block transcriptional activity [20]. We observed that overexpression of HNF1A-AS1 along with iCRT14 decreased the protein levels of CMYC, OCT4, SOX2, and NANOG (Fig. 4I and Supplementary Fig. 2E, F). qRT-PCR results showed that iCRT14 similarly reversed the transcriptional activation of target genes downstream of the Wnt/β-catenin pathway by HNF1A-AS1 (Fig. 4J, K). Certainly, the ability of HNF1A-AS1 to promote GC cell proliferation was also inhibited in response to iCRT14 (Fig. 4L, M). These results suggest that the role of HNF1A-AS1 in maintaining the stemness of GCSCs depends on the Wnt/β-catenin pathway.

### HNF1A-AS1 upregulates the expression of β-catenin by adsorbing miR-150-5p

We aimed to investigate how HNF1A-AS1 increased the expression of β-catenin. The intracellular localization of lncRNAs is critical to their function [21]. Our previous studies indicated that HNF1A-AS1 was present in both the nucleus and cytoplasm [11]. Our experimental data suggested that HNF1A-AS1 may function through post-transcriptional regulation by acting as a molecular sponge for miRNAs, which play a crucial role in this process. This allows lncRNAs to act as ceRNAs in the cytoplasm [22–24].

**Fig. 5:**
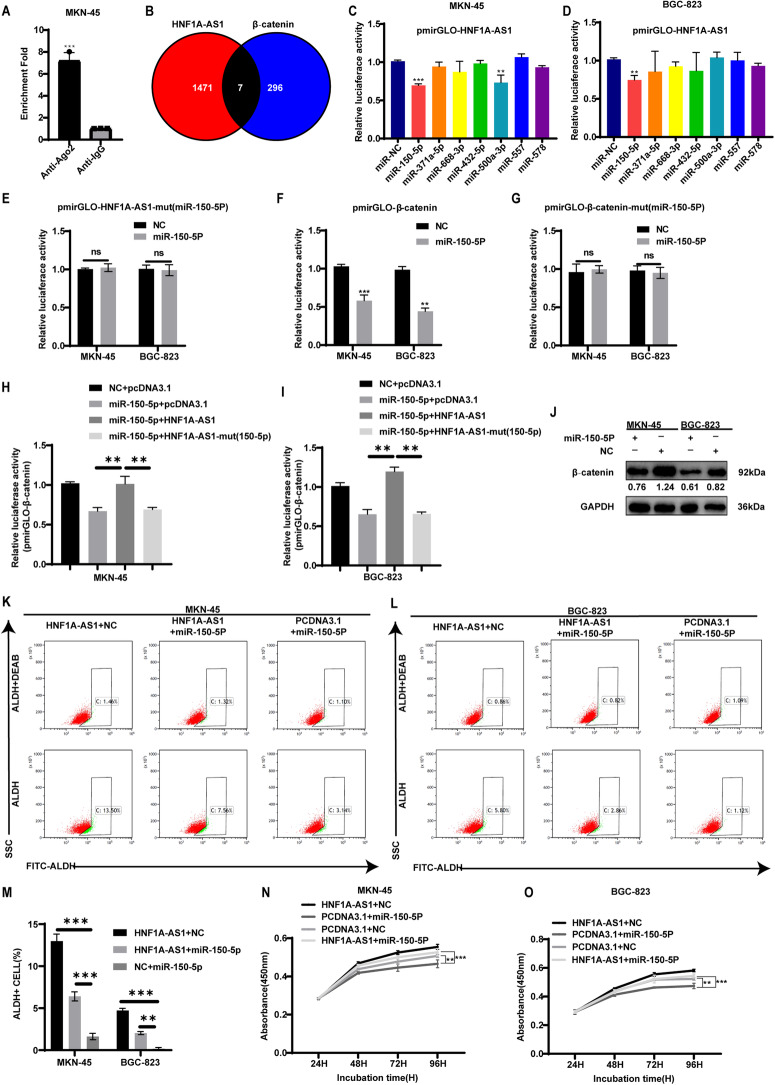
HNF1A-AS1 upregulates the expression of β-catenin by adsorbing miR-150-5p. **A** RIP assay results were analyzed using PCR to confirm that HNF1A-AS1 could combine with Ago2 protein, and IgG was used as the control protein (*n* = 3). **B** A Venn diagram depicting 7 miRNAs that predicted to target both HNF1A-AS1 and β-catenin by RegRNA 2.0 algorithms. **C**, **D** Relative luciferase activity of pmirGLO-HNF1A-AS1 in GC cells co-transfected with the 7 miRNAs (*n* = 3). Relative luciferase activity of pmirGLO-HNF1A-AS1-mut (miR-150-5p) (**E**), pmirGLO-β-catenin (**F**) and pmirGLO-β-catenin-mut (miR-150-5p) (**G**) in GC cells co-transfected with miR-150-5p (*n* = 3). **H**, **I** Relative luciferase activity of pmirGLO-β-catenin in GC cells when overexpressing miR-150-5p, HNF1A-AS1 or HNF1A-AS1-mut (miR-150-5p) (*n* = 3). **J** Western blotting assay was used to determine the effect of miR-150-5p on the expression of β-catenin. **K**, **L** Representative flow cytometric plot of ALDH+ cells in MKN-45 and BGC-823 transfected with HNF1A-AS1, miR-150-5p and PCDNA3.1. **M** Bar graph shows quantification of ALDH+ cells in GC cells (*n* = 3). **N**, **O** The cell viability was determined with CCK8 assays when overexpressing miR-150-5p or HNF1A-AS1 (*n* = 3). Data are representative as the mean ± SD. Two-tailed unpaired Student’s t-test (**A**, **E**, **F**, **G**), one-way ANOVA with Tukey’s multiple-comparison test (**C**, **D**, **H**, **I**, **M**), two-way ANOVA test (**N**, **O**). *P < 0.05; **P < 0.01; ***P < 0.001.

To confirm the binding of HNF1A-AS1 and miRNAs, we performed RNA immunoprecipitation (RIP) assays using the MKN-45 cell line. The results showed that the Ago2 protein bound to HNF1A-AS1 and mediated the interaction with miRNAs in the cytoplasm (Fig. 5A). We predicted which miRNAs could bind to the 3′ UTR region of HNF1A-AS1 and β-catenin using the software RegRNA 2.0 (http://regrna.mbc.nctu.edu.tw). By taking the intersection, we identified seven miRNAs that could bind to both HNF1A-AS1 and β-catenin (Fig. 5B). Luciferase assays demonstrated that miR-150-5p reduced pmirGLO-HN1A-AS1 luciferase activity in GC cells (Fig. 5C, D). We further mutated the binding site of miR-150-5p on HNF1A-AS1, and the luciferase assay confirmed that miR-150-5p bound to the corresponding site on HNF1A-AS1 (Fig. 5E).

Using the same approach, we found that miR-150-5p also bound to the 3′ UTR region of β-catenin (Fig. 5F, G). The results of luciferase assay showed that overexpression of HNF1A-AS1 reversed the inhibitory effect of miR-150-5p on pmirGLO-β-catenin 3′ UTR luciferase activity in GC cells, whereas HNF1A-AS1-mut (miR-150-5p) failed to reverse the effect of miR-150-5p (Fig. 5H, I). Overexpressing miR-150-5P in GC cells resulted in reduced β-catenin protein expression (Fig. 5J and Supplementary Fig. 3A). Rescue assays confirmed that miR-150-5p reversed the effect of HNF1A-AS1 and suppressed the stemness of GCSCs (Fig. 5K–M). MiR-150-5P also significantly weakened the proliferative capacity of GC cells overexpressing HNF1A-AS1 (Fig. 5N, O). Our findings confirmed a ceRNA network of HNF1A-AS1, miR-150-5P, and β-catenin in GC.

### CMYC promotes HNF1A-AS1 expression by directly binding to the promoter of HNF1A-AS1

**Fig. 6:**
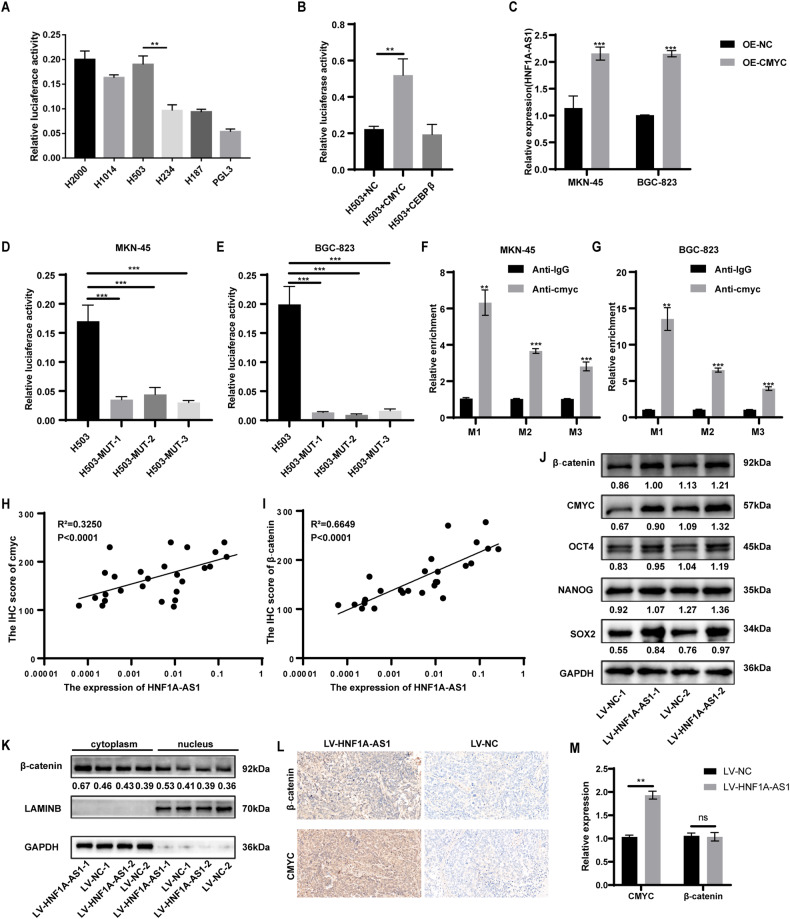
CMYC promotes HNF1A-AS1 expression by directly binding to the promoter of HNF1A-AS1. **A** Transcriptional activity analysis of the potential HNF1A-AS1 promoter fragments in MKN-45 cells (*n* = 3). **B** Relative luciferase activity assay showed that CMYC observably increased promoter activities (*n* = 3). **C** qRT-PCR assay indicated CMYC promoted the expression level of HNF1A-AS1 in GC cells (n = 3). **D**, **E** Relative luciferase activity of the HNF1A-AS1 promoter was decreased when the three presumed HNF1A-AS1 binding site was mutated (*n* = 3). **F**, **G** ChIP-qPCR analysis indicated higher fold enrichment of promoter amplicons of CMYC in anti-CMYC antibody group than that of IgG group in GC cells, indicating that CMYC could directly bind to HNF1A-AS1 promoter (*n* = 3). M1, M2 and M3 respectively represent primer that covered the CMYC-binding sites. **H**, **I** Gastric cancer tissue samples were immunohistochemically stained for CMYC and β-catenin for expression scoring. Correlation analysis was performed with HNF1A-AS1 expression levels (*n* = 26). **J** Western blotting analyses of stemness-related gene expression in mouse xenograft tumors. **K** Spatial localization of β-catenin in mouse xenograft tumor cells determined by Western blotting. **L** Representative images of IHC for β-catenin and CMYC in tumors from xenograft mice. Original magnification, ×400. **M** Relative quantification of mRNA expression of β-catenin and CMYC in tumors from xenograft mice (*n* = 3). Data are representative as the mean ± SD. One-way ANOVA with Tukey’s multiple-comparison test (**A**, **B**, **D**, **E**), two-tailed unpaired Student’s t-test (**C**, **F**, **G**, **M**), Spearman’s correlation (**H**, **I**). *P < 0.05; **P < 0.01; ***P < 0.001.

We conducted further investigations to determine the reasons behind the increased expression of HNF1A-AS1 in GCSCs. We constructed sequences of several deletions in the HNF1A-AS1 promoter region in the pGL3-basic vector to evaluate the transcriptional regulation of transcription factors targeting HNF1A-AS1. A luciferase activity assay revealed a significant decrease in fluorescence values in the −503 to −234 bp region, indicating that this segment was the core promoter region with a possible transcription factor binding site (Fig. 6A). The online software JASPAR (http://jaspar.genereg.net/) showed that CMYC and CEBPβ were potential transcription factors. The dual-luciferase reporter assay demonstrated that CMYC enhanced the activity of the HNF1A-AS1 promoter (Fig. 6B). The qRT-PCR analysis confirmed that the expression of HNF1A-AS1 was elevated after overexpression of CMYC in GC cell lines (Fig. 6C). We introduced three point-mutations to investigate the contribution of the three putative CMYC-binding sites to the regulation of the HNF1A-AS1 promoter. Each of the GC cell lines was transfected with the three mutant plasmids and subjected to a luciferase assay. Each of the three mutant vectors reduced promoter activity relative to the wild-type control, indicating that the CMYC-binding site cluster was responsible for HNF1A-AS1 transcription (Fig. 6D, E). These results indicate that the CMYC-binding site is essential for HNF1A-AS1 transcription.

A ChIP assay was performed to determine whether CMYC specifically interacted with the HNF1A-AS1 promoter. Using an anti-CMYC antibody, the promoter amplicon of HNF1A-AS1 was enriched at the three binding sites in GC cells (Fig. 6F, G). We performed CMYC and β-catenin immunohistochemical staining in gastric cancer tissue specimens for expression scoring. A correlation analysis was performed with HNF1A-AS1 expression levels. The results showed that the expression of CMYC (*R*^2^ = 0.3250, *P* < 0.0001) and β-catenin (R^2^ = 0.6649, *P* < 0.0001) in gastric cancer tissues showed a positive correlation with the expression of HNF1A-AS1 (Fig. 6H, I).

To confirm whether the mechanism by which HNF1A-AS1 maintained stemness in xenograft tumor-bearing mice was consistent with the results observed in GC cells, we collected the tumors deriving from 2 × 10^5^ dilution in vivo experiments and investigated the protein levels of β-catenin, CMYC, OCT4, SOX2, and NANOG in xenograft tumors formed by LV-HNF1A-AS1-transfected cells compared with control LV-NC-transfected cells. We found that LV-HNF1A-AS1-transfected tumors had higher levels of β-catenin, CMYC, OCT4, SOX2, and NANOG than controls (Fig. 6J and Supplementary Fig. 3B, C). We also investigated the localization of β-catenin in the xenograft tumors and observed an increase in its nuclear localization in the LV-HNF1A-AS1 group (Fig. 6K and Supplementary Fig. 3D, E). Immunohistochemical assays showed increased expression of β-catenin and CMYC in LV-HNF1A-AS1-transfected tumors (Fig. 6L). qRT-PCR assays showed increased mRNA levels of *CMYC* in these tumors, whereas there was no significant difference in *β-catenin* mRNA levels (Fig. 6M).

## Discussion

CSCs exhibit stem cell properties such as self-renewal, differentiation, and drug resistance [25, 26]. In GC patients with metastasis and recurrence, GCSCs are the main culprits [27]. However, the exact mechanisms by which GCSCs maintain self-renewal and stemness remain mostly unknown. A previous study from our group showed that HNF1A-AS1 was associated with lymph node metastasis in patients and served as a biological marker to predict lymph node metastasis in patients [28]. However, the function played by HNF1A-AS1 in GCSC was not clear. Our study aimed to investigate how HNF1A-AS1 regulated the self-renewal and stemness of GCSCs via the Wnt/β-catenin pathway cascade. We found that HNF1A-AS1 was abnormally expressed in gastric tumor stem cells and that its overexpression promoted the stemness of GC cells, as evidenced by the monoclonal formation assay, tumor cell microsphere formation assay, and ALDH enzyme activity assay. LncRNAs can regulate the function of CSCs by mediating transcription factors, classical stem cell-associated pathways, and miRNAs that maintain stem cells. However, we do not yet know how lncRNAs specifically regulate the stemness of gastric tumor cells.

In additional experiments, we found that although HNF1A-AS1 affected the protein level of β-catenin, a marker associated with gastric tumor stem cells, it did not significantly regulate its transcriptional level, indicating a potential post-transcriptional mechanism of action. In recent years, the ceRNA mechanism by which lncRNAs affect the expression level of miRNA target genes has become a popular topic of research [29–31]. For example, GCMA was shown to act as a ceRNA to sponge miR-124 and miR-34a, upregulating the expression of slug and snail [32]. We proposed that HNF1A-AS1 may upregulate the expression of β-catenin by absorbing miRNAs. Our previous studies showed that HNF1A-AS1 was present in both the nucleus and cytoplasm, making it possible that HNF1A-AS1 acted through a ceRNA mechanism. We explored the potential interaction between HNF1A-AS1, miRNAs, and β-catenin through bioinformatics, luciferase, and RIP analyses. We determined the direct binding ability of the predicted miRNA to the 3′ UTR region of the full-length HNF1A-AS1 transcript and the corresponding mRNA of *β-catenin*. Our experiments showed that HNF1A-AS1 acted as a ceRNA by absorbing miR-150-5p, which protected the expression of the target gene *β-catenin* from inhibition. This ultimately promoted β-catenin entry into the nucleus to activate the Wnt/β-catenin pathway and promote the stemness of gastric tumor cells.

In recent years, numerous studies have indicated a potential association between lncRNA and the Wnt/β-catenin pathway in CSCs. For example, HNF1A-AS1 expression was an independent prognostic factor affecting the overall survival of osteosarcoma patients, and the knockdown of HNF1A-AS1 reduced β-catenin expression [33]. It has also been shown that the knockdown of HNF1A-AS1 in esophageal squamous carcinoma inhibited the epithelial-mesenchymal transition (EMT) and stemness by regulating the miR-298/TCF4 axis [34]. A study on colorectal cancer showed that reduced expression of HNF1A-AS1 inhibited Wnt/β-catenin activity by downregulating the expression of β-catenin, cyclin D1, and CMYC [35]. These studies suggested that the classical Wnt/β-catenin pathway may play a role in regulating stemness in various tumor cells, with varying specific mechanisms. The transcription activation of stemness-regulated related target genes, such as *CMYC*, *SOX4*, *CCND1*, *CCND2*, and *TCF7*, occurs downstream of the Wnt/β-catenin pathway [36–38]. In non-small cell lung cancer, miR-150-5p exerted anti-cancer effects by targeting HMGA2 and Wnt/β-catenin signaling [39]. In glioma, miR-150-5p inhibited tumor progression by regulating stem cell-like properties through modulation of the Wnt/β-catenin pathway [40]. Although these studies reported an inhibitory effect of miR-150-5p on the Wnt/β-catenin pathway, the specific mechanism of action of miR-150-5p had not been elucidated. Our results showed that in GC, miR-150-5p bound to the 3’ UTR region of *β-catenin* and inhibited the translation process of β-catenin protein. The present study’s results indicated that HNF1A-AS1 promoted GC cell stemness through the post-transcriptional regulation of β-catenin, resulting in elevated β-catenin expression in GC cells, thereby causing abnormal activation of the Wnt/β-catenin pathway. Therefore, HNF1A-AS1 can be targeted to intervene in the self-renewal of gastric tumor stem cells.

CMYC is a downstream transcription factor of the Wnt/β-catenin pathway [41, 42]. Based on UCSC and JASPAR databases, we predicted that the promoter of HNF1A-AS1 contained multiple binding sites for CMYC. Subsequently, dual-luciferase assays and ChIP assays confirmed that CMYC acted as a transcriptional activator that directly bound to the promoter of HNF1A-AS1, inducing its expression. Furthermore, CMYC was upregulated in GC cells overexpressing HNF1A-AS1.

**Fig. 7:**
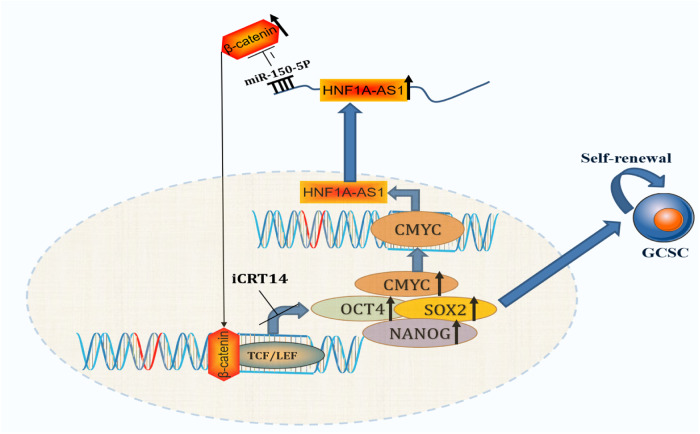
Schematic representation of the functional role of HNF1A-AS1 in regulating GCSCs. HNF1A-AS1 together with CMYC and miR-150-5p formed a positive feedback loop to activate β-catenin expression in GC.

Overall, HNF1A-AS1 activated the Wnt/β-catenin pathway and upregulated CMYC expression by increasing β-catenin expression and promoting its nuclear translocation. Subsequently, the transcription factor CMYC further increased the expression of HNF1A-AS1 (Fig. 7). Our findings in gastric cancer reveal a novel axis involving miR-150-5P and the Wnt/β-catenin signaling pathway, which could serve to maintain the stemness of GCSC and is expected to be a new therapeutic target in the future.

### Supplementary information

Supplementary data

Western blot

## Data Availability

All remaining data are available within the article and supplementary files, or available from the corresponding author upon request.
