# Supplementary material for: CMYC-initiated HNF1A-AS1 overexpression maintains the stemness of gastric cancer cells
Source: Cell Death Dis. 2024 Apr 23;15(4):288. doi: 10.1038/s41419-024-06673-y (PMC11039746; doi:10.1038/s41419-024-06673-y)
Supplement: Supplementary file 1 — Supplementary data [file 41419_2024_6673_MOESM1_ESM.docx]

**
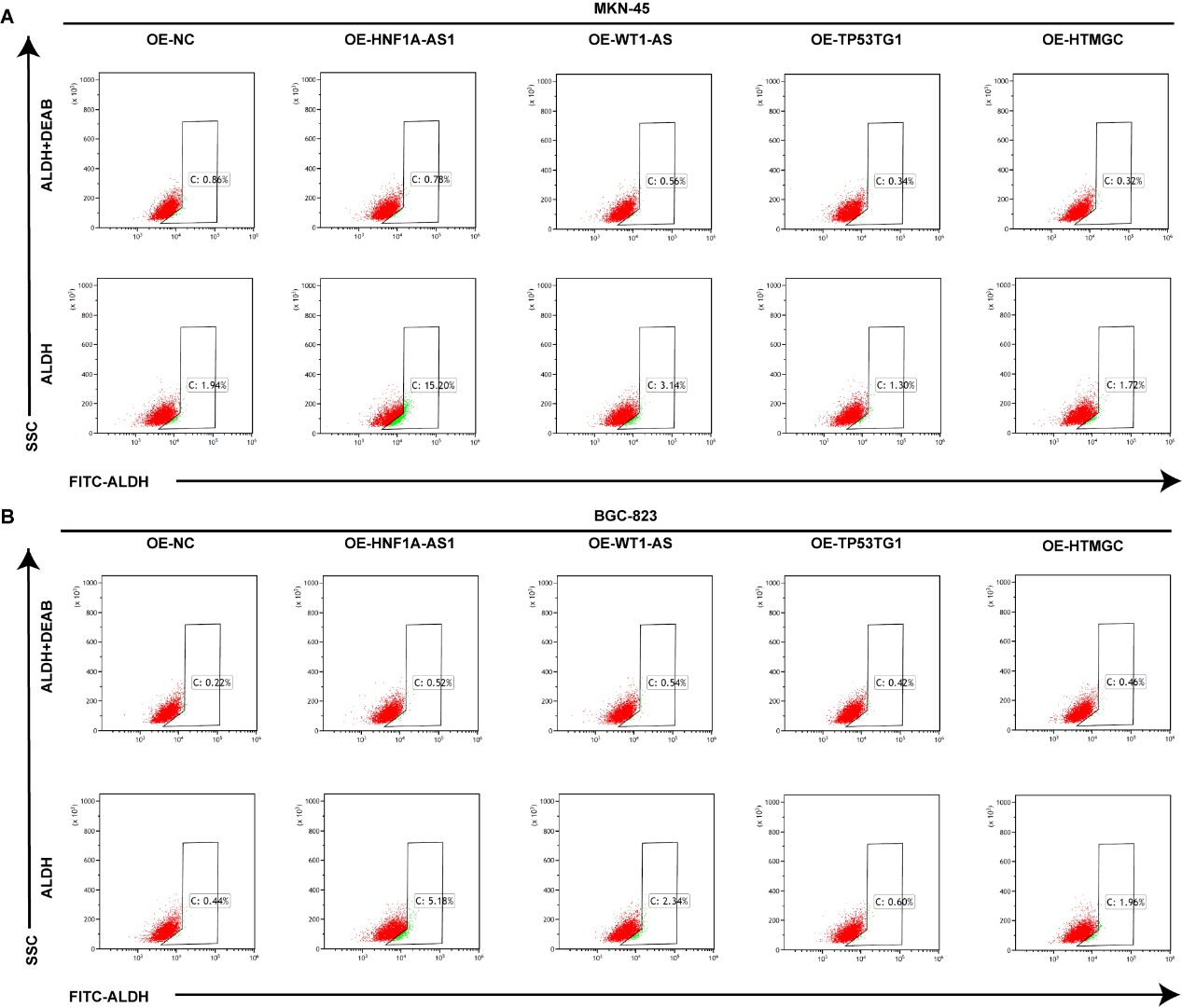
**

**Supplementary Fig. 1** HNF1A-AS1 promotes ALDH enzyme activity in GC cells

(A, B) Representative flow cytometric plot of ALDH+ cells in MKN-45 and BGC-823 transfected with HNF1A-AS1, WT1AS, TP53TG1, HTMGC and PCDNA3.1. Numbers are showing ratio of ALDH+ cells to viable cells.

**
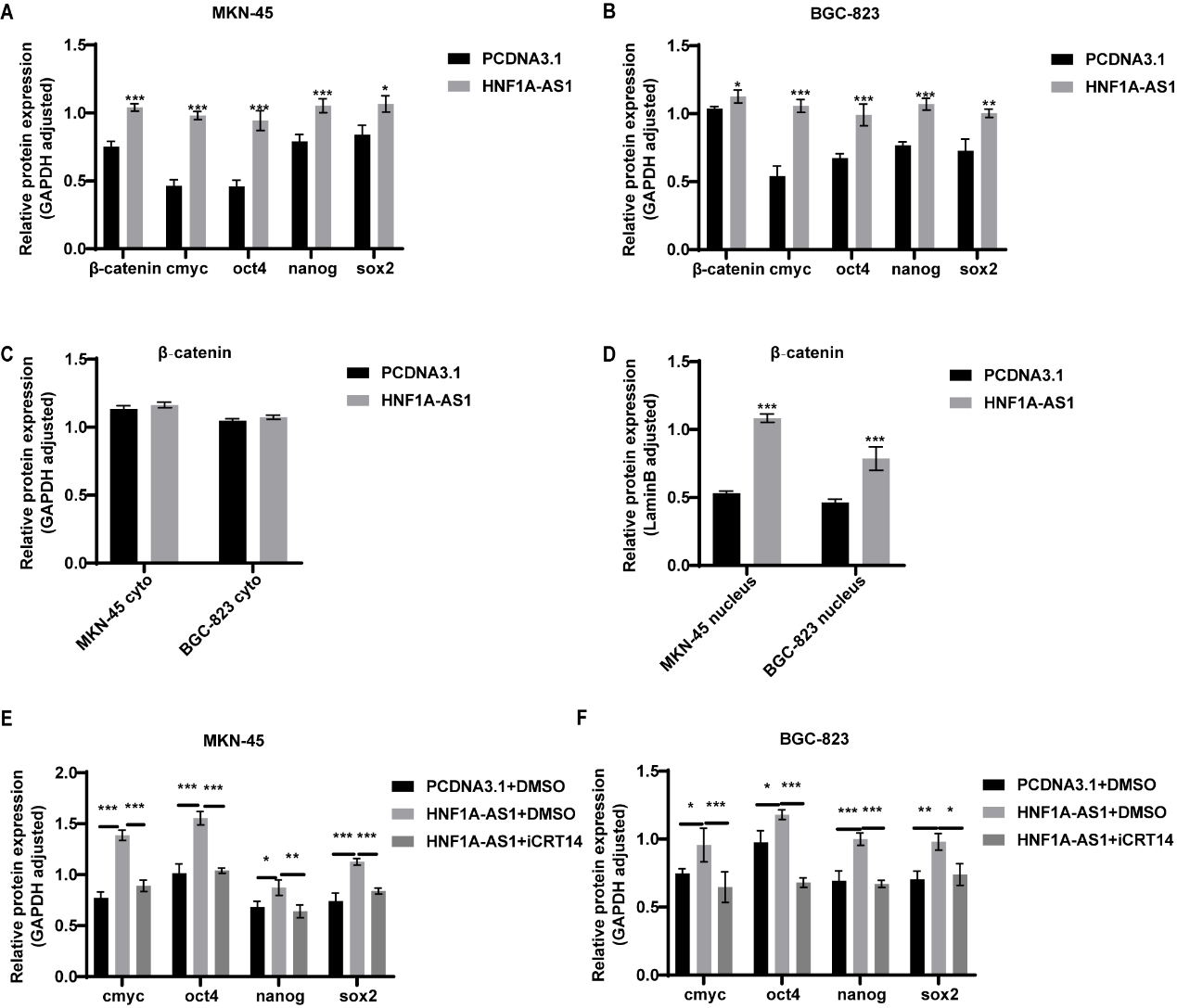
**

**Supplementary Fig. 2** Relative quantitative analysis of proteins.

(A, B) Quantification shows the relative expression levels of target genes of the Wnt pathway associated with cell stemness after overexpression of HNF1A-AS1 (n=3). (C, D) Spatial localization of B was detected after transfection of GC cells with HNF1A-AS1 using nucleoplasmic separation and western blotting relative quantitative analysis (n=3). (E, F) The antagonistic effect of iCRT14 on the action of HNF1A-AS1 on the activation of the WNT pathway was analyzed by western blotting relative quantification (n=3). Data are representative as the mean ± SD. Two-tailed unpaired Student’s t test (A, B, C and D), one-way ANOVA with Tukey’s multiple-comparison test (E and F). *P < 0.05; **P < 0.01; ***P < 0.001.


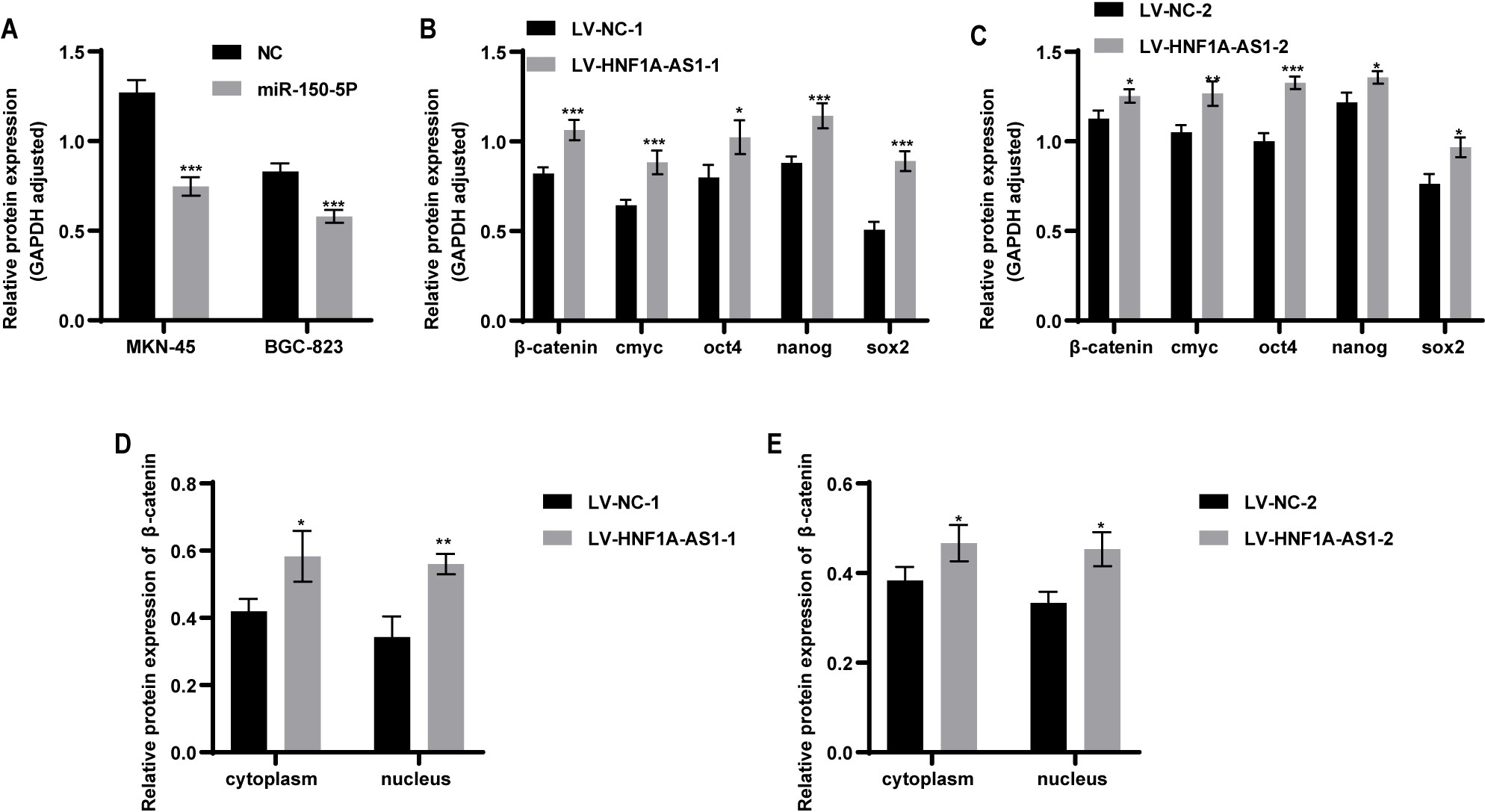


**Supplementary Fig. 3** Relative quantitative analysis of proteins.

(A) Relative quantitative analysis of the effect of miR-150-5P in GC cells on the expression level of β-catenin (n=3). (B, C) Relative quantitative analysis of WNT pathway stemness-related gene activation levels in mouse xenograft tumors with varying levels of HNF1A-AS1 expression (n=3). (D, E) Relative quantitative analysis of the spatial localization of β-catenin in mouse xenograft tumors with different levels of HNF1A-AS1 expression (n=3). Data are representative as the mean ± SD. Two-tailed unpaired Student’s t test (A–E). *P < 0.05; **P < 0.01; ***P < 0.001.

| **Supplementary, Table S1. Sequences of RT-qPCR primers** | |
| --- | --- |
| HNF1A-AS1-F | AGACAAGAGTTTGCCACATTGC |
| HNF1A-AS1-R | ACAAAGCAAGACCCCCATCTC |
| OCT4(POU5F1)-F | TCAGGAGATATGCAAAGCAGAA |
| OCT4(POU5F1)-R | TTGCCTCTCACTCGGTTCTC |
| SOX2-F | ACATGAACGGCTGGAGCAA |
| SOX2-R | GTAGGACATGCTGTAGGTGGG |
| NANOG-F | AGATGCCTCACACGGAGACT |
| NANOG-R | GTTTGCCTTTGGGACTGGTG |
| CMYC-F | CCCCTACCCTCTCAACGACA |
| CMYC-R | CTTCTTGTTCCTCCTCAGAGTCG |
| CTNNB1(β-Catenin)-F | GCTGCAACTAAACAGGAAGGG |
| CTNNB1(β-Catenin)-R | CCCACTTGGCAGACCATCAT |
