# Supplementary material for: CMYC-initiated HNF1A-AS1 overexpression maintains the stemness of gastric cancer cells
Source: Cell Death Dis. 2024 Apr 23;15(4):288. doi: 10.1038/s41419-024-06673-y (PMC11039746; doi:10.1038/s41419-024-06673-y)
Supplement: Supplementary file 2 — Western blot [file 41419_2024_6673_MOESM2_ESM.pdf]

Figure 4A（The biological replicate-1, which is presented in the figure.）

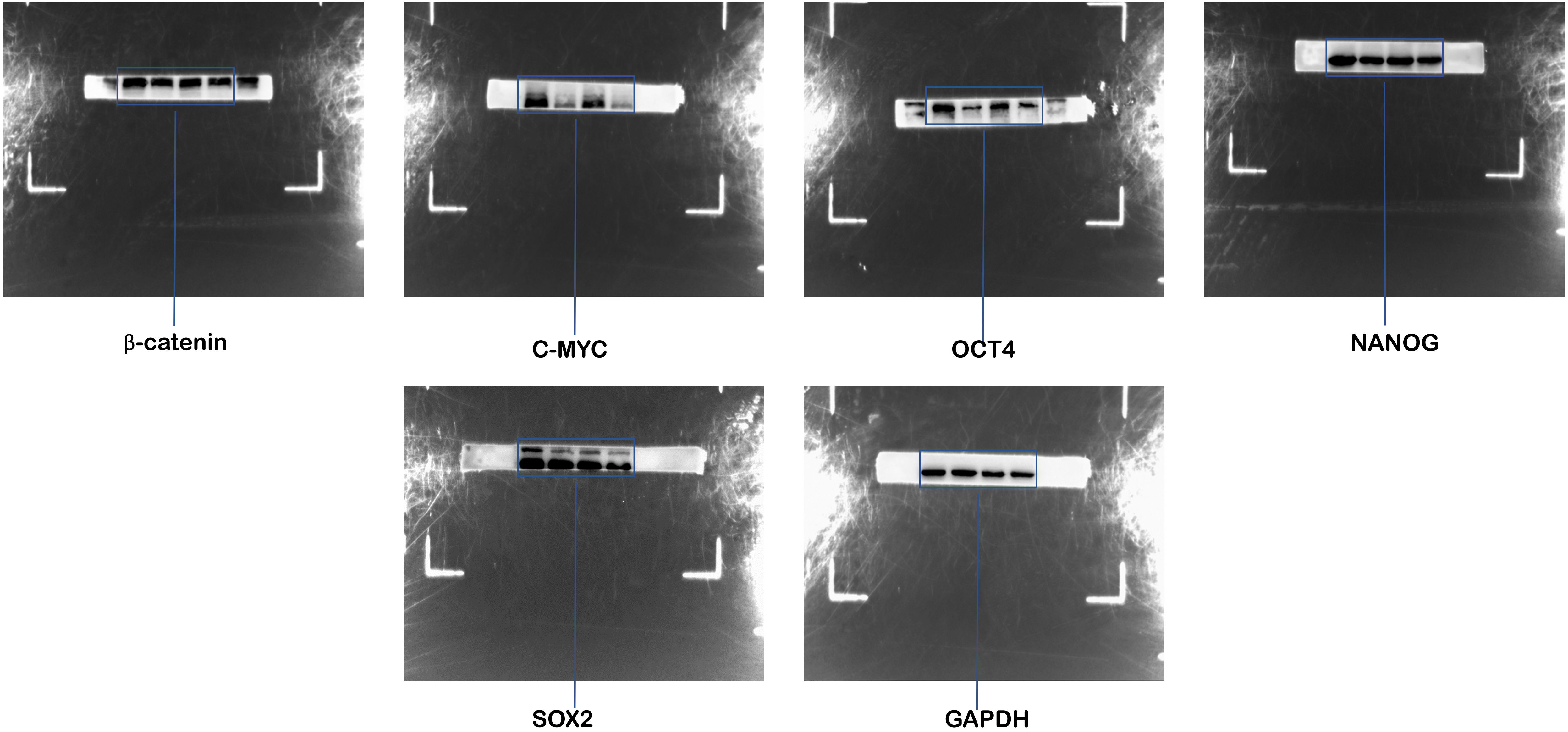

Figure 4A（The biological replicate-2.）

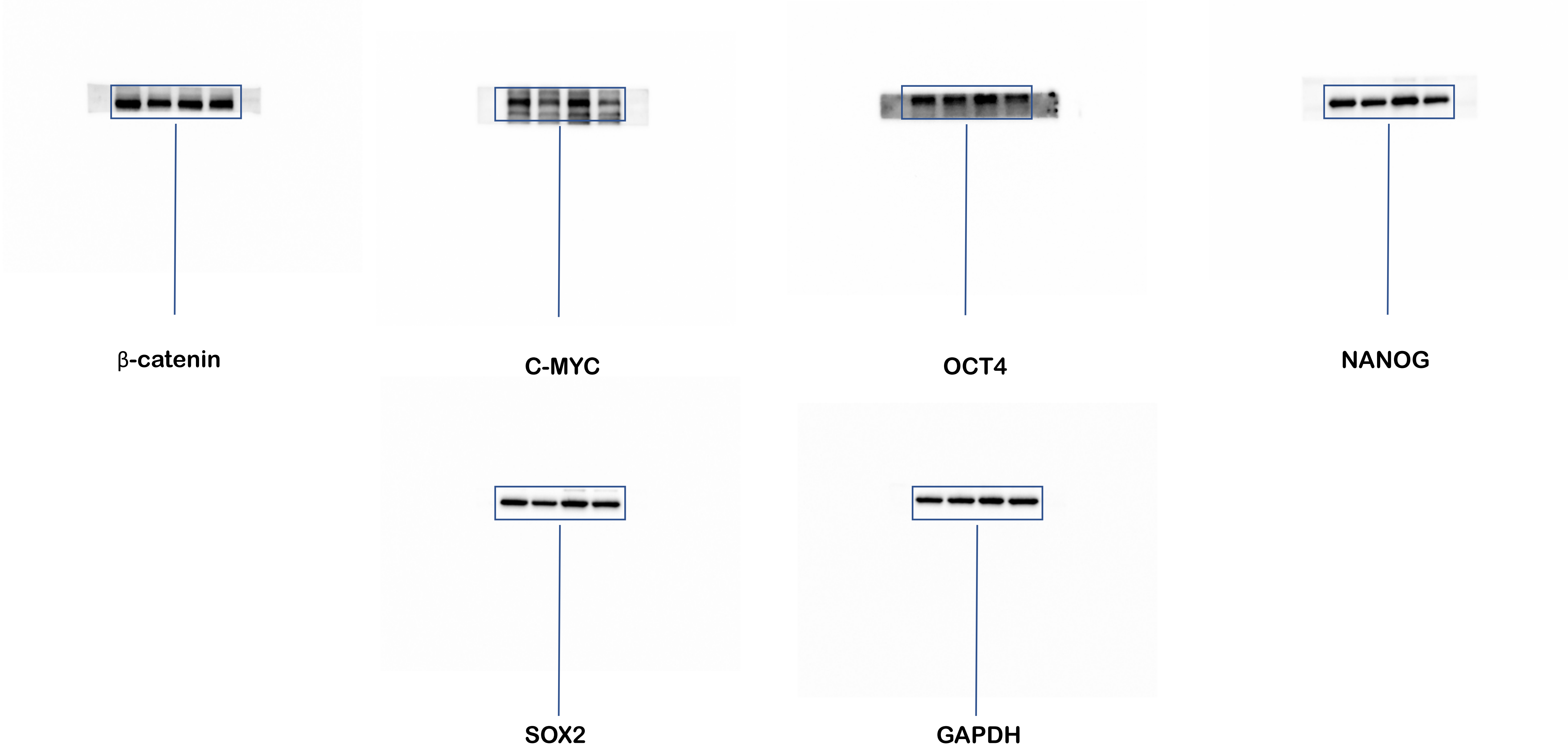

Figure 4A（The biological replicate-3.）

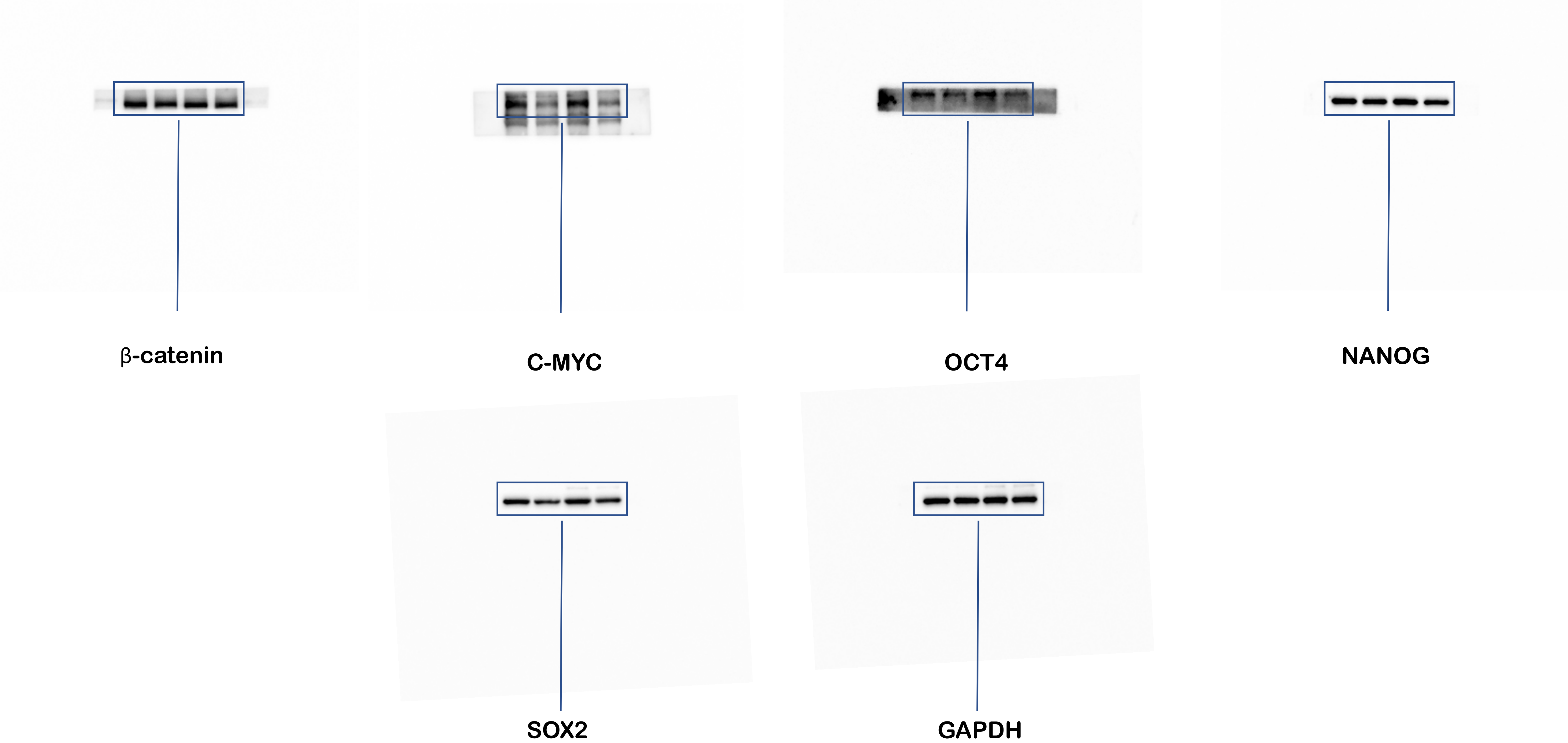

Figure 4H（The biological replicate-1, which is presented in the figure.）

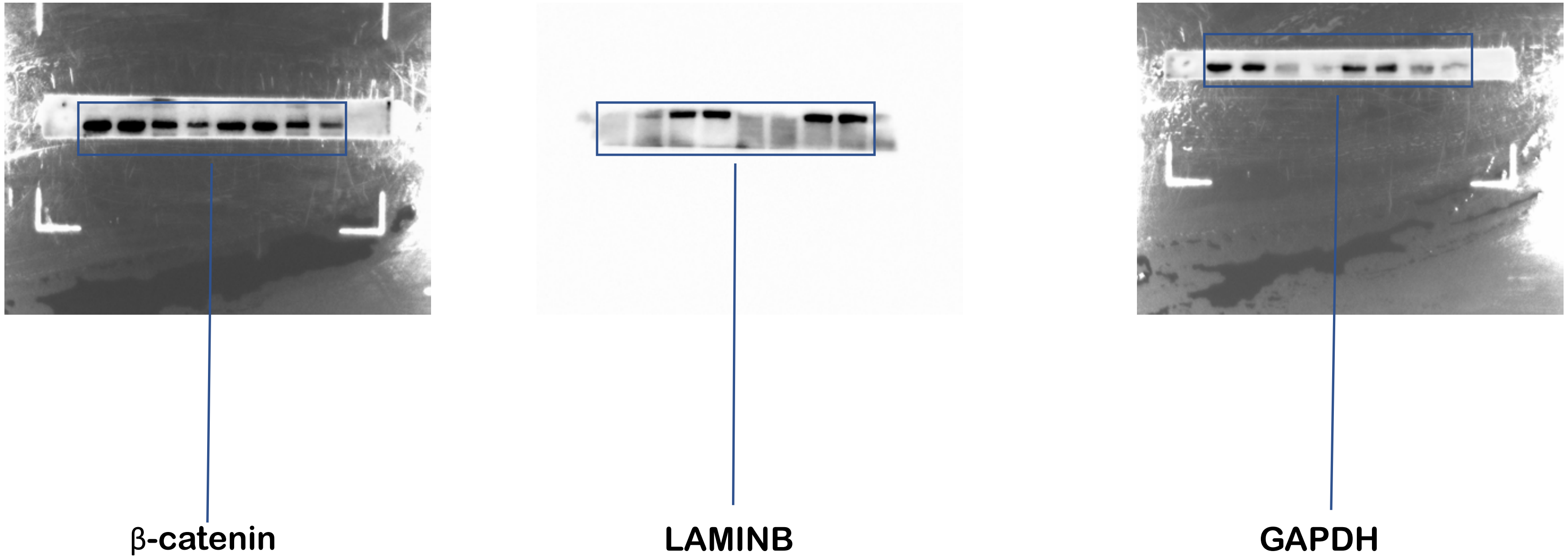

Figure 4H（The biological replicate-2.）

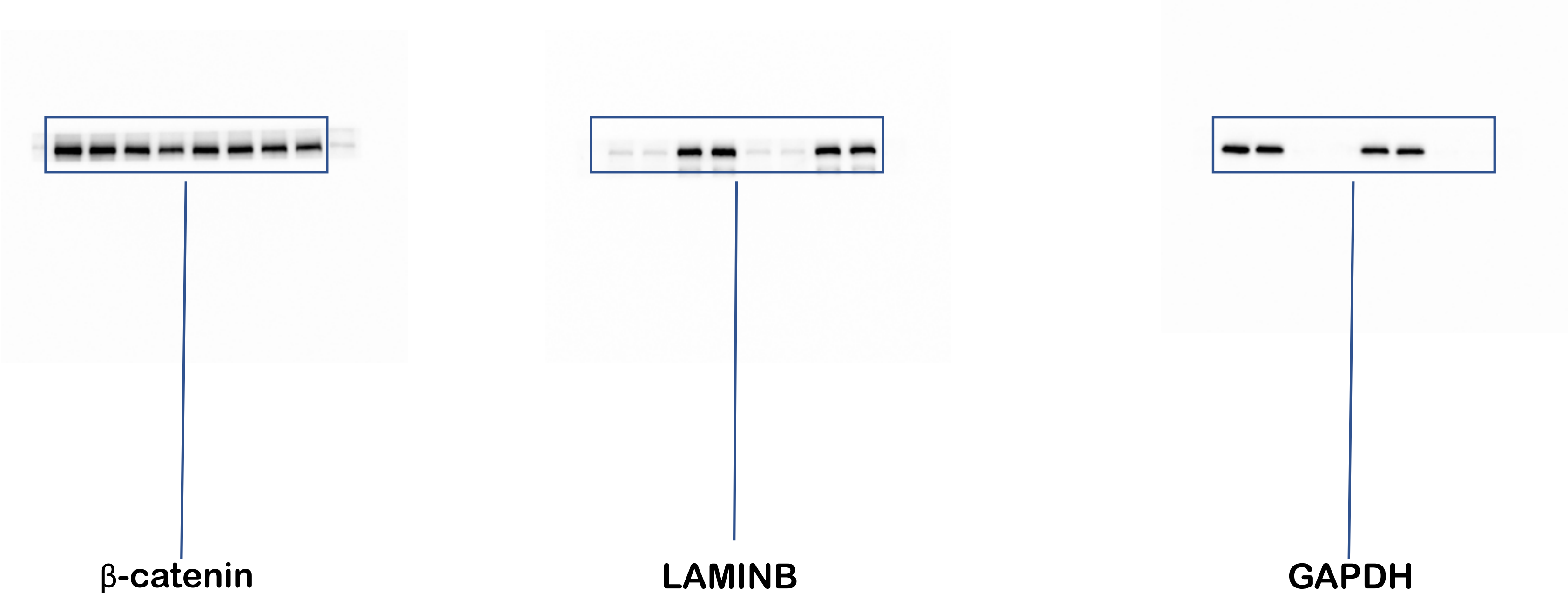

Figure 4H（The biological replicate-3.）

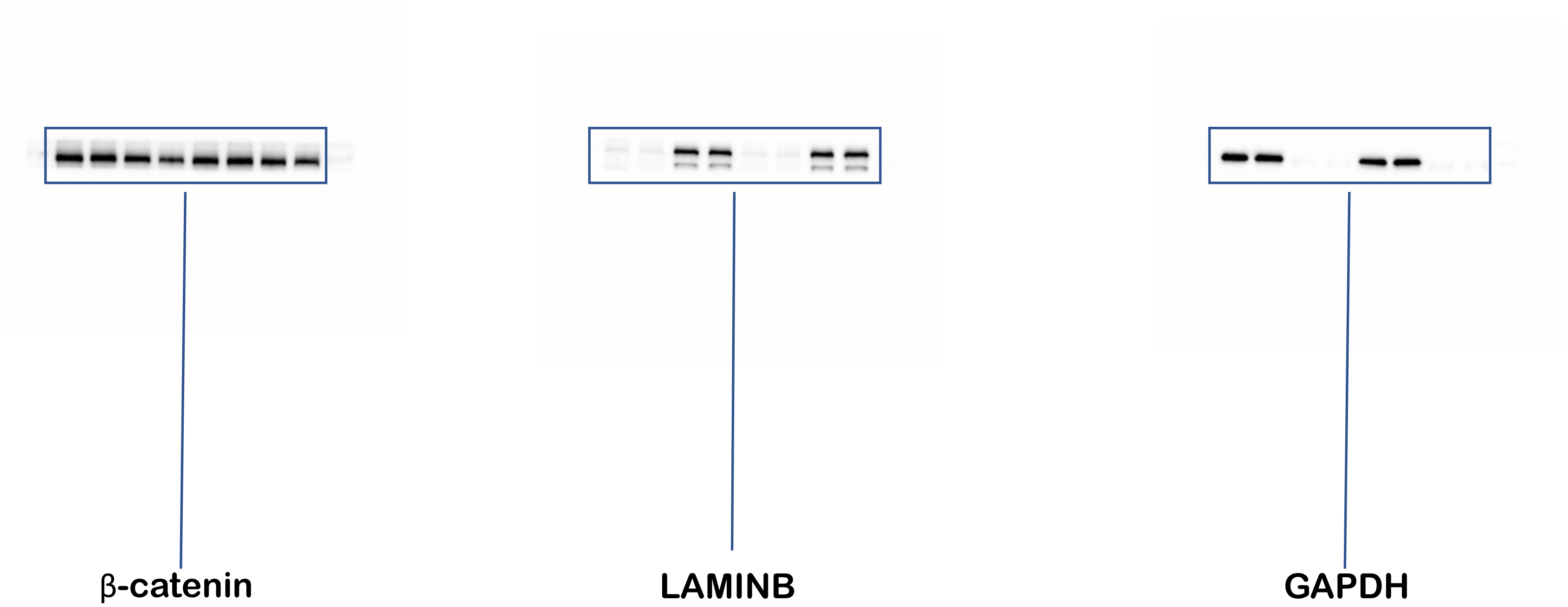

Figure 4I (The biological replicate-1, which is presented in the figure.)

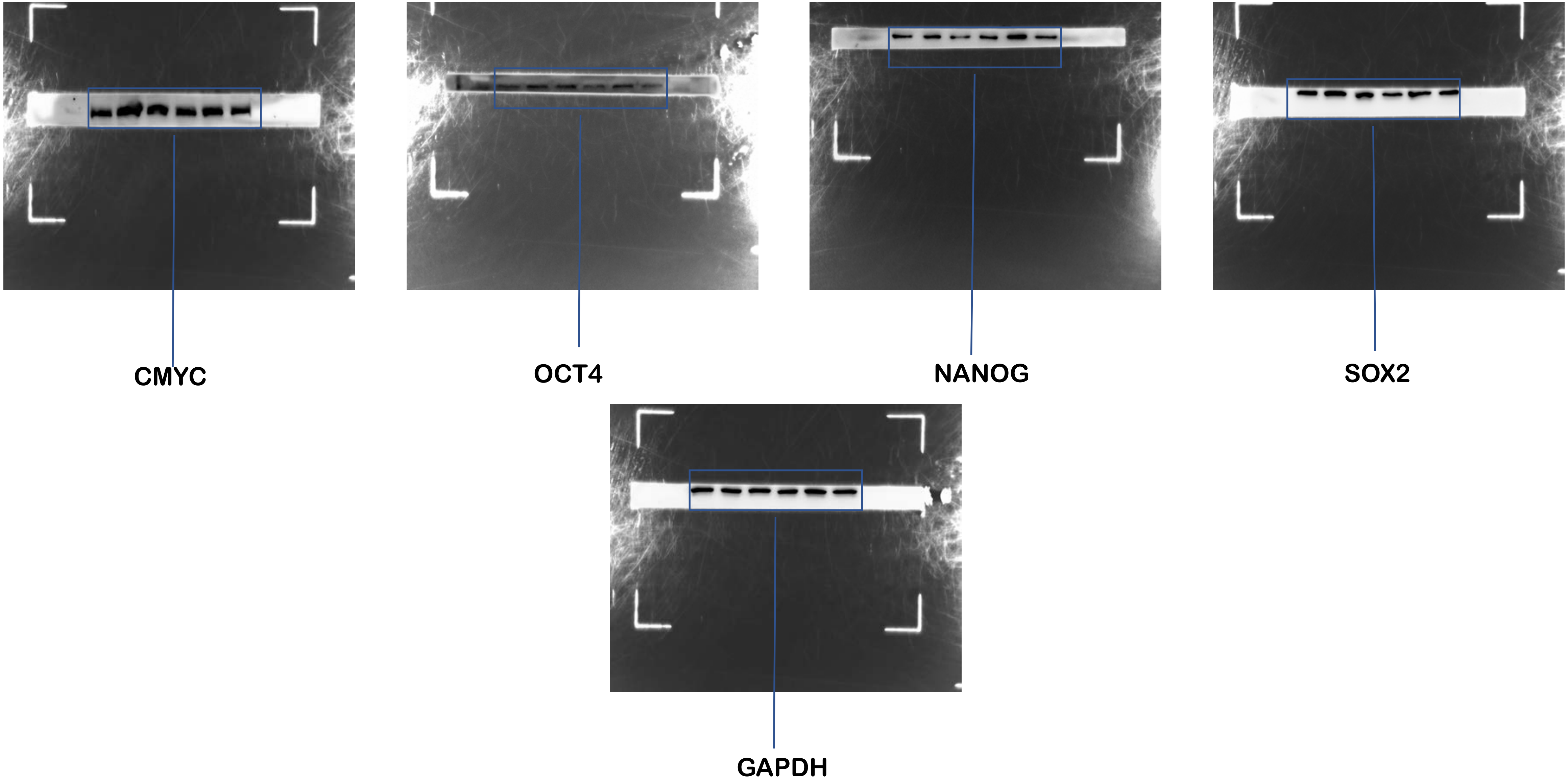

Figure 4I (The biological replicate-2.)

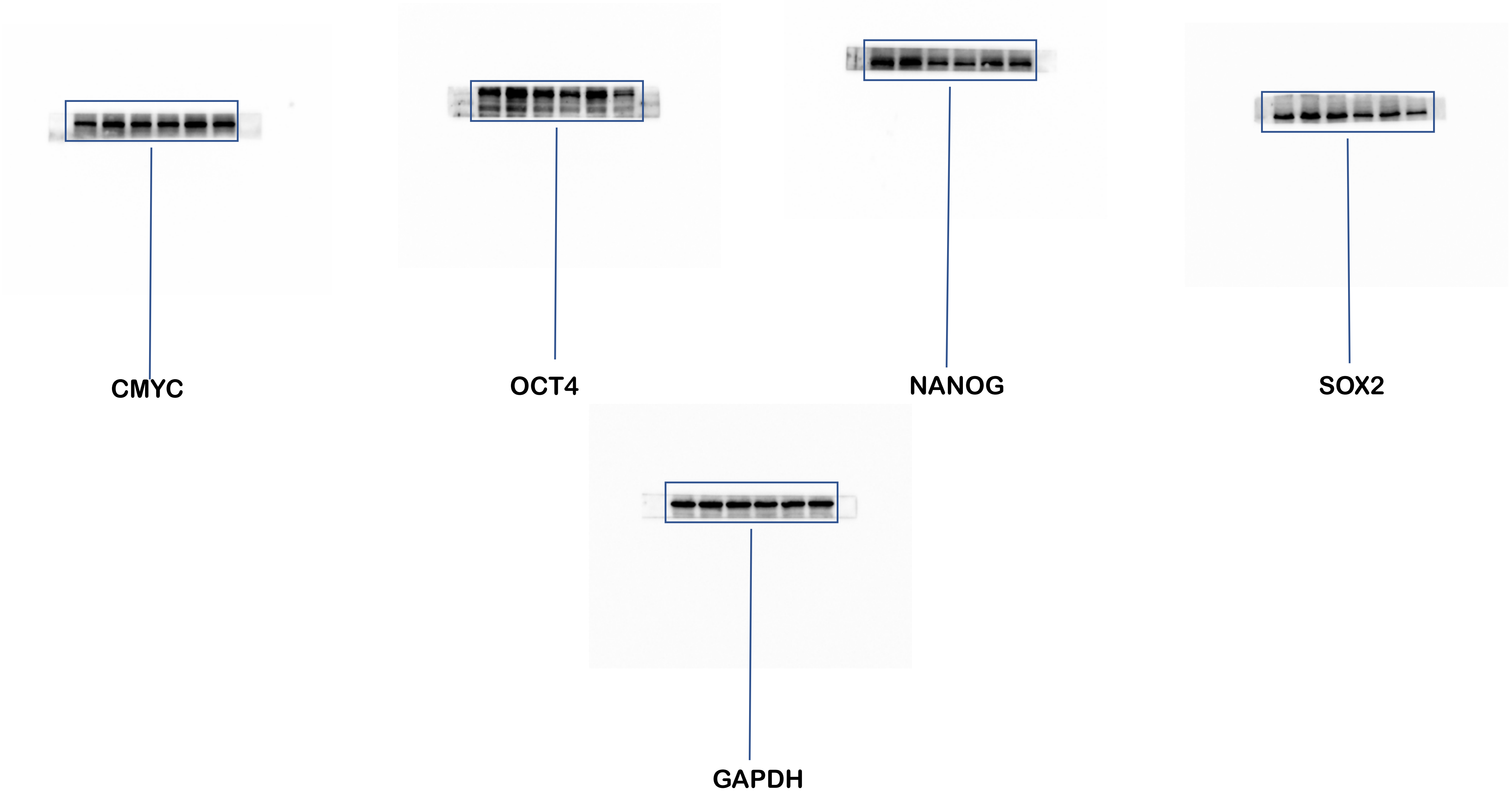

Figure 4I (The biological replicate-3.)

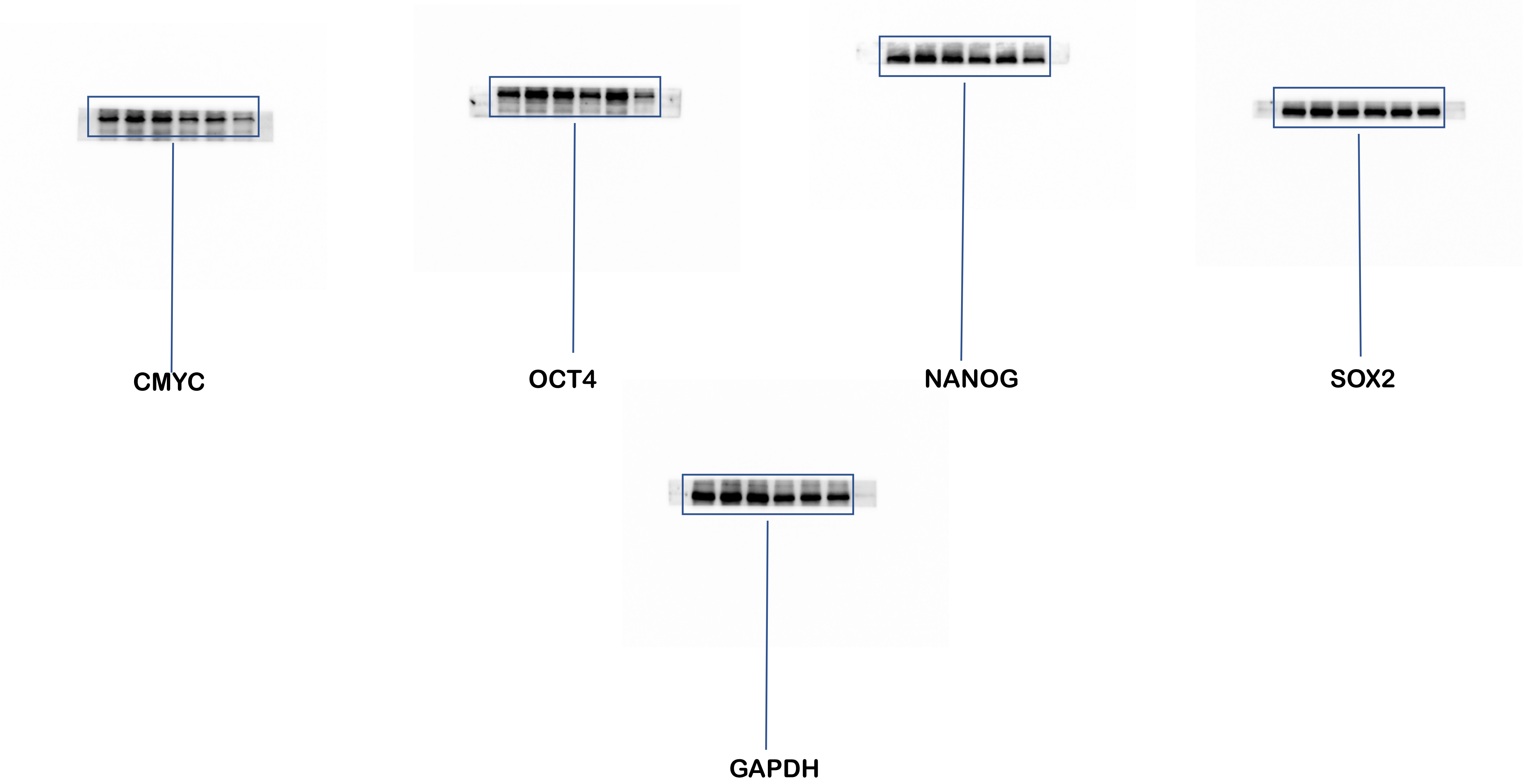

Figure 5J (The biological replicate-1, which is presented in the figure.)

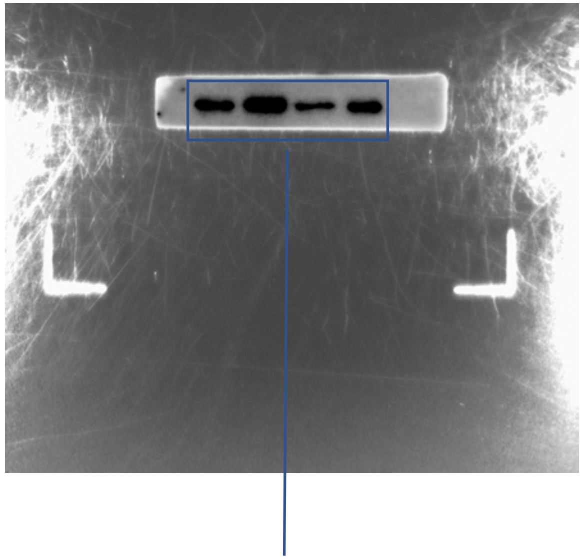

β-catenin

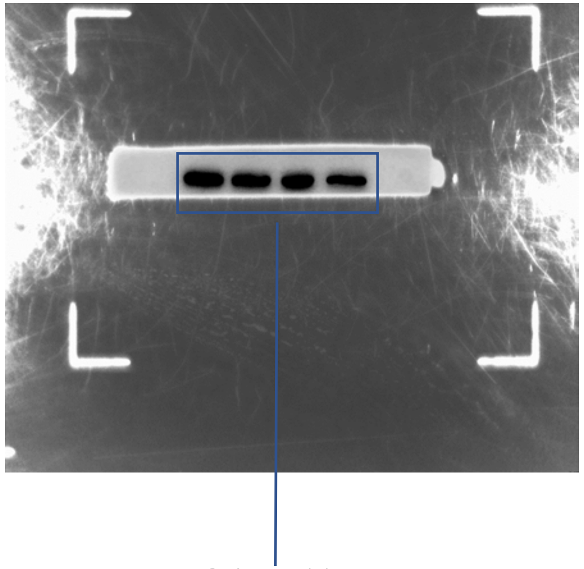

GAPDH

Figure 5J (The biological replicate-2.)

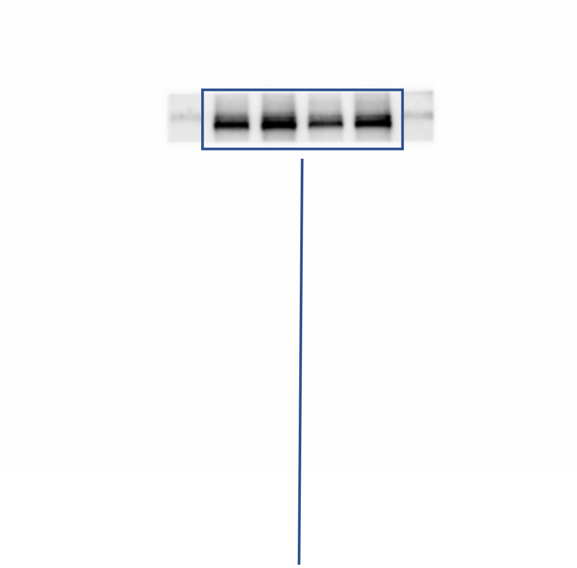

β-catenin

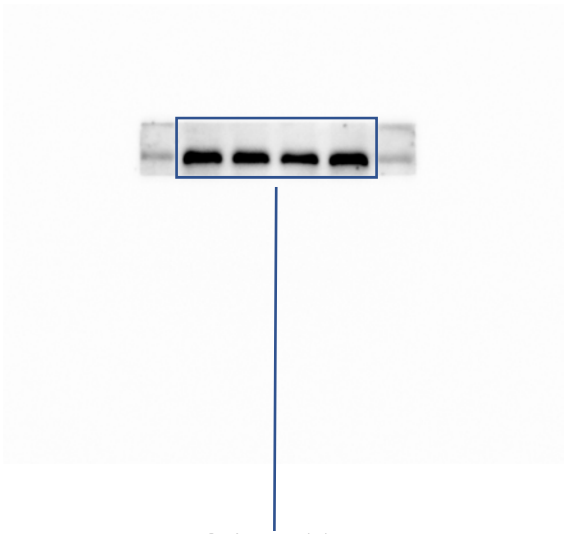

GAPDH

Figure 5J (The biological replicate-3.)

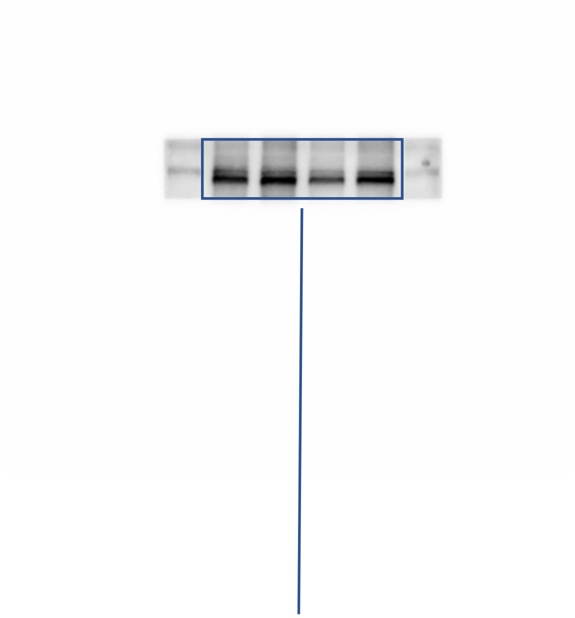

β-catenin

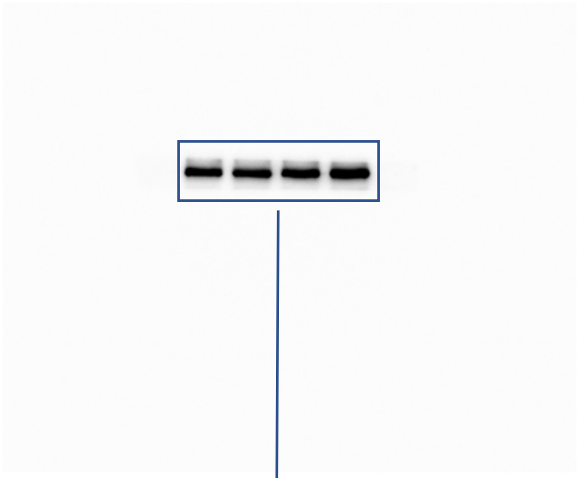

GAPDH

Figure 6J (The biological replicate-1, which is presented in the figure.)

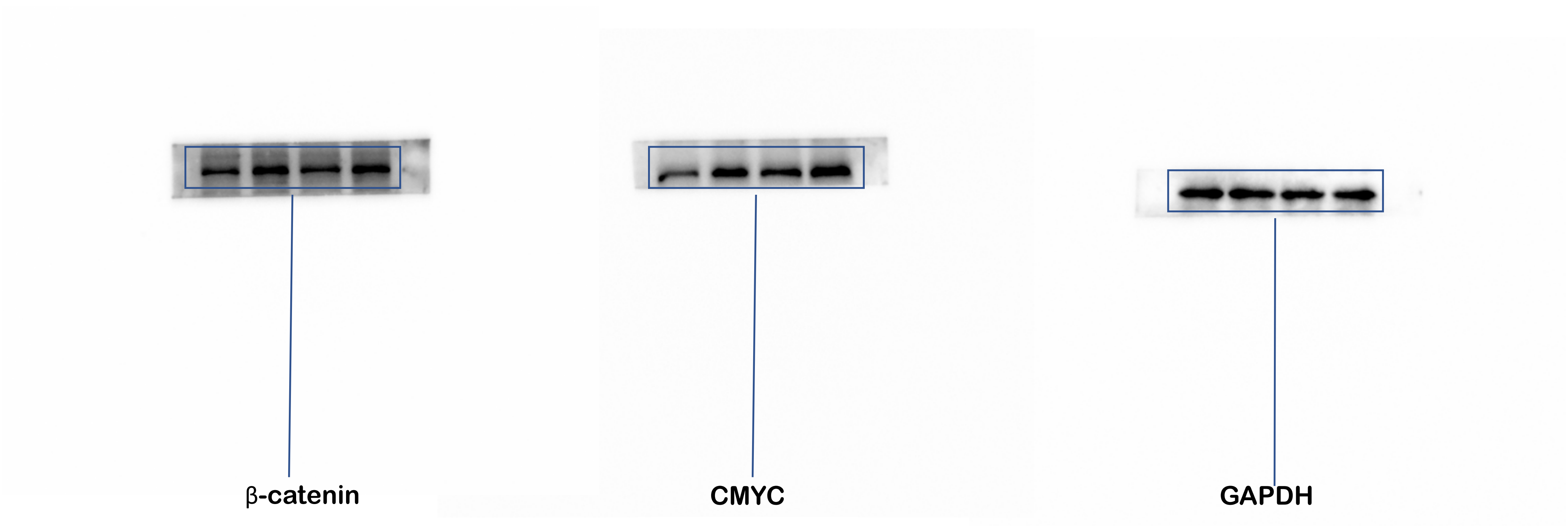

Figure 6J (The biological replicate-1, which is presented in the figure.)

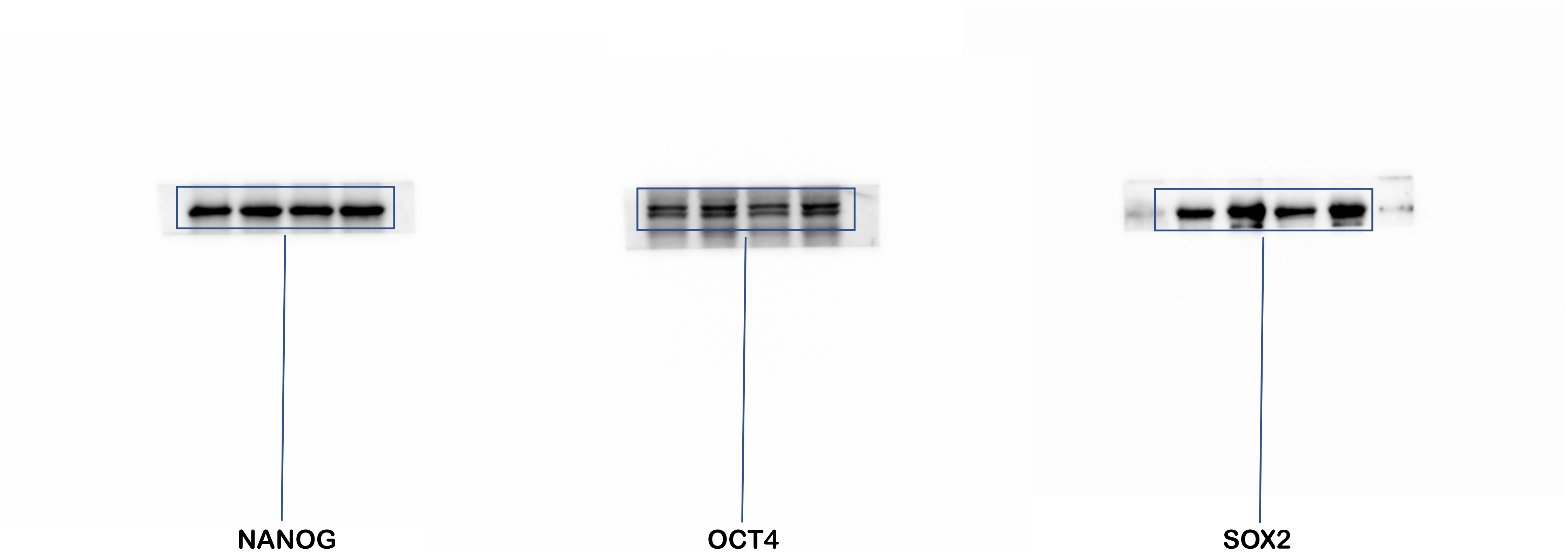

Figure 6J (The biological replicate-2.)

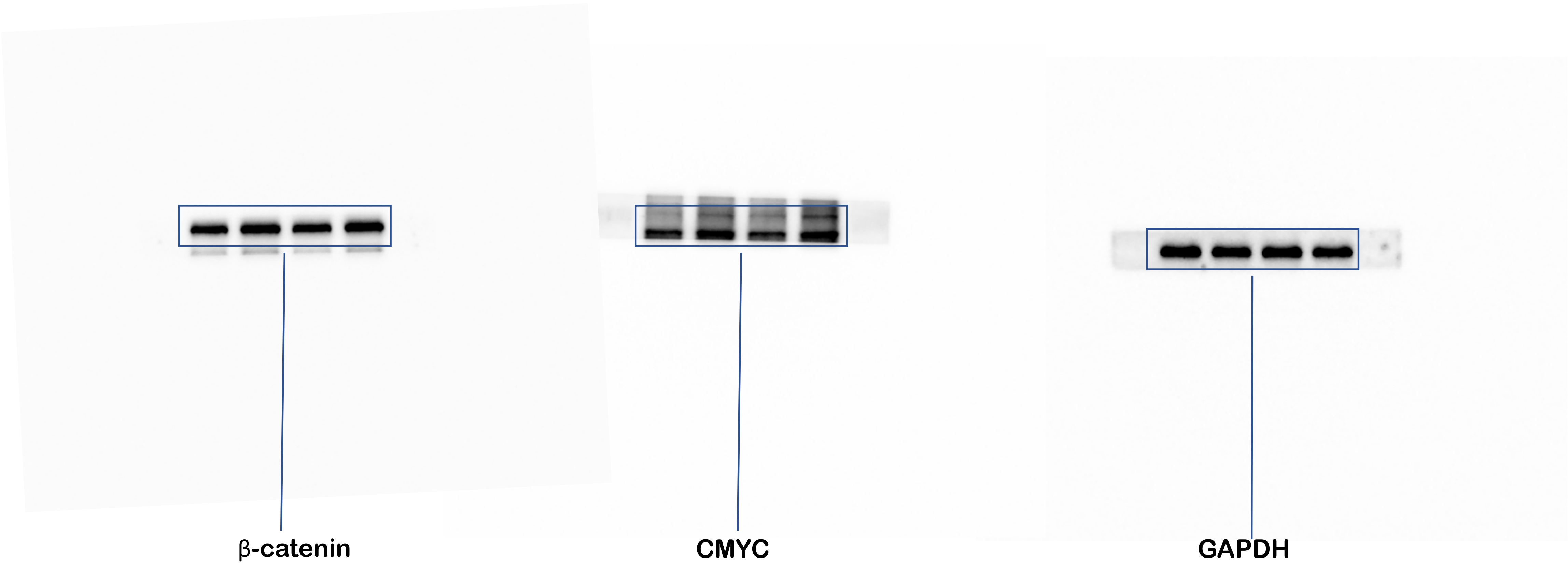

Figure 6J (The biological replicate-2.)

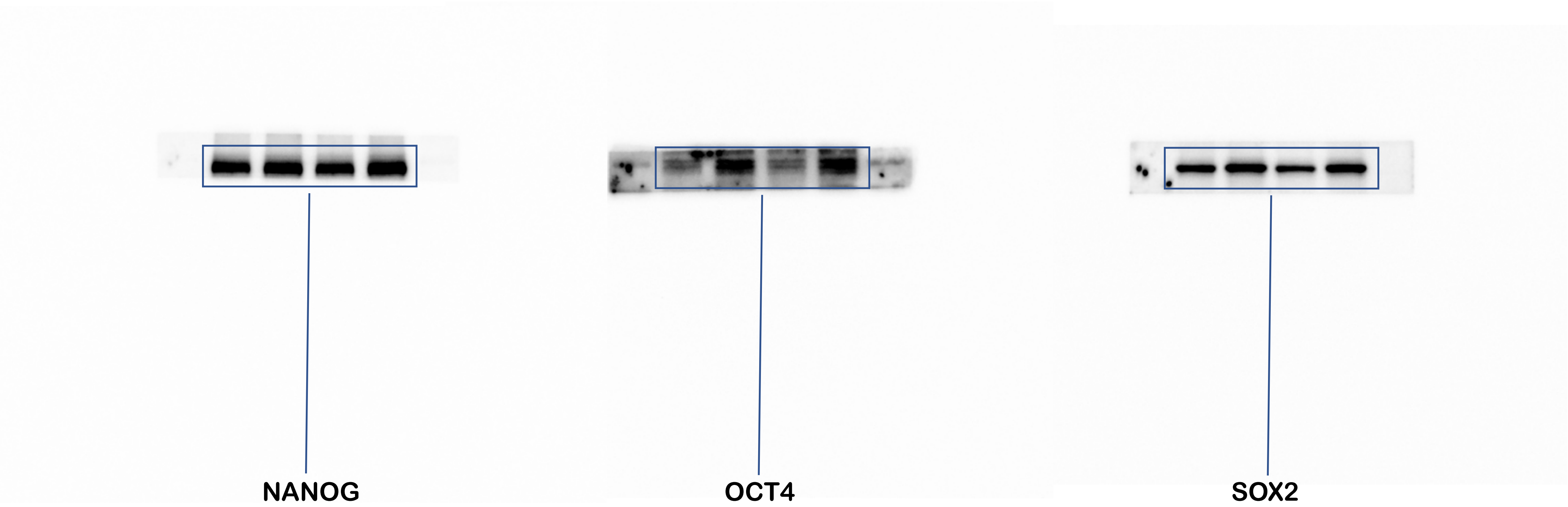

Figure 6J (The biological replicate-3.)

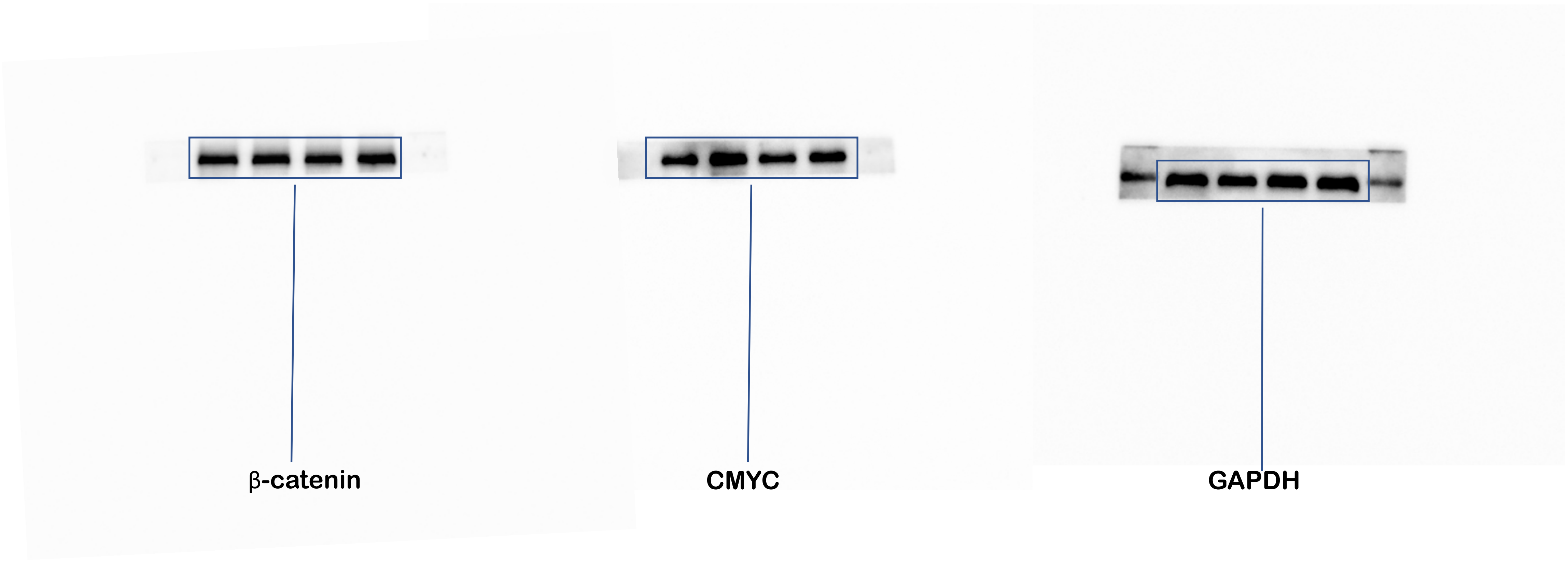

Figure 6J (The biological replicate-3.)

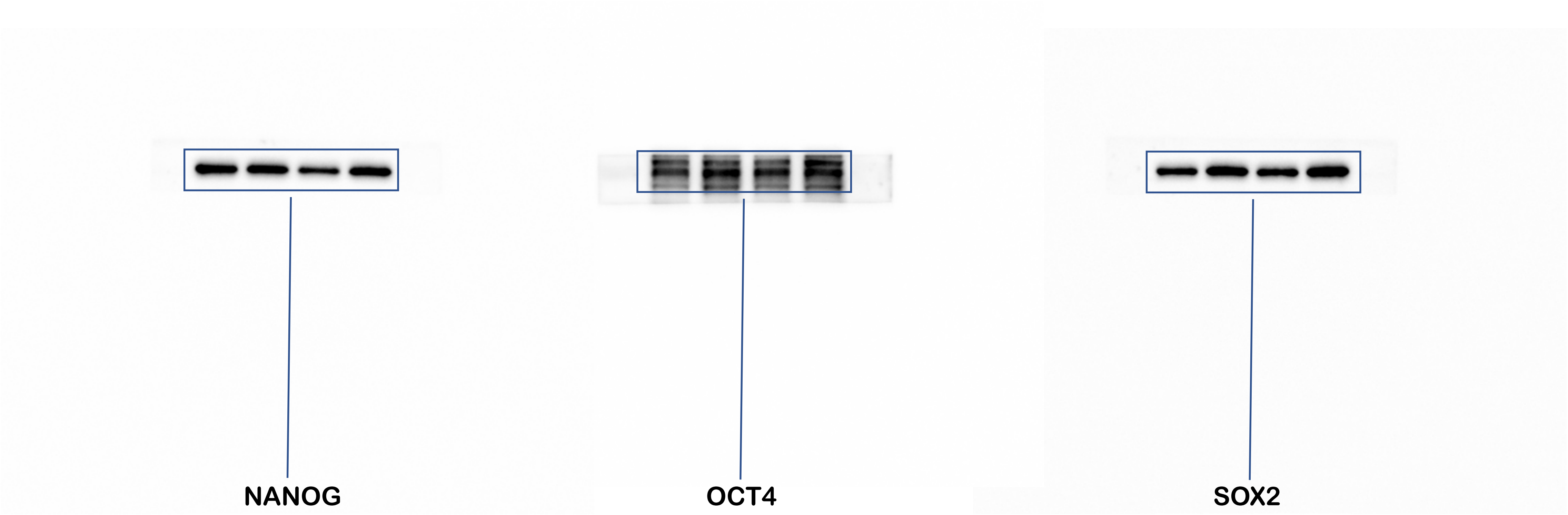

Figure 6K (The biological replicate-1, which is presented in the figure.)

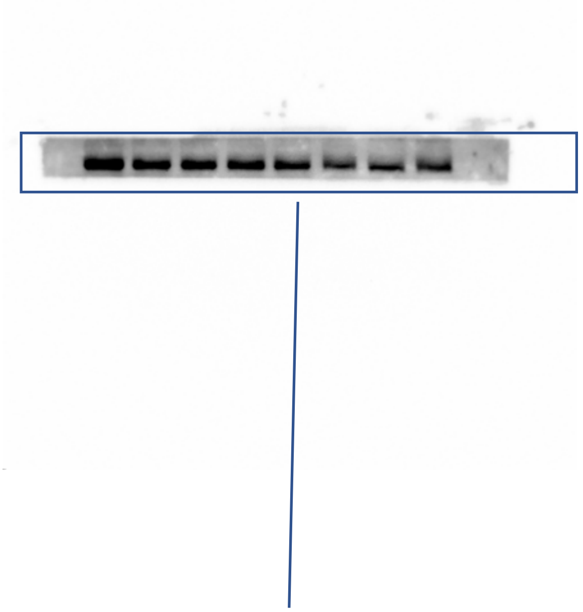

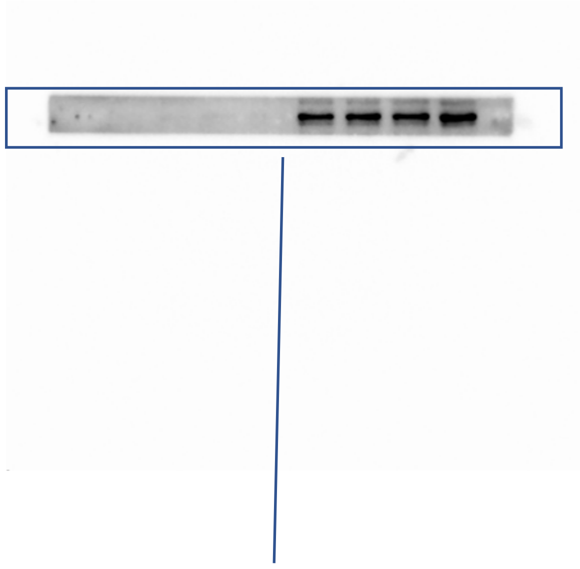

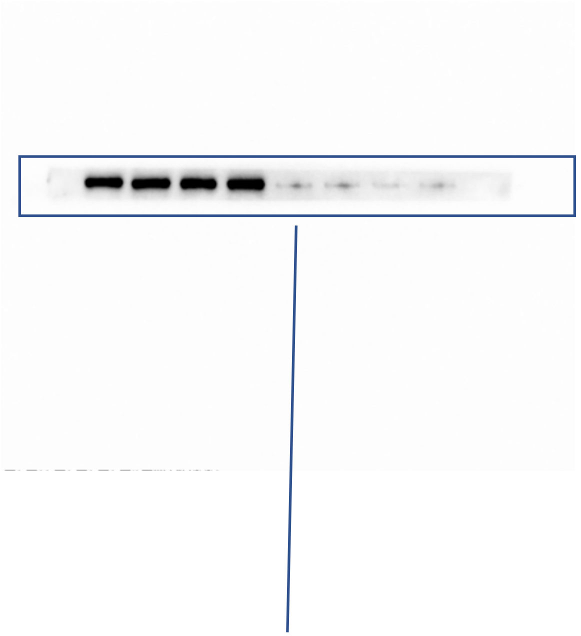

Figure 6K (The biological replicate-2.)

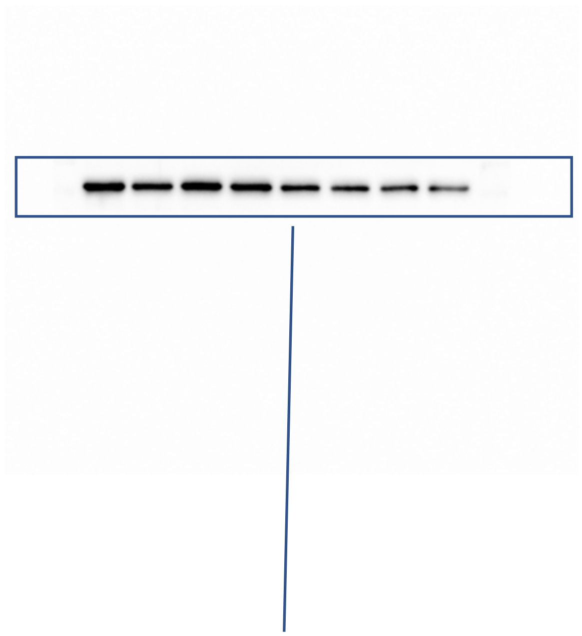

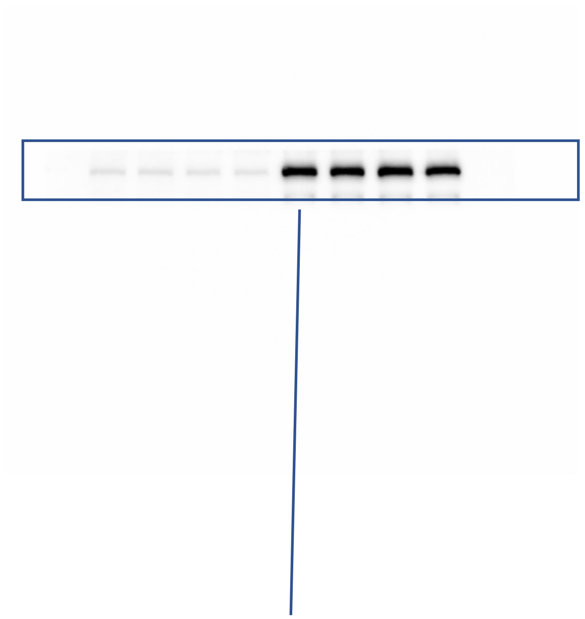

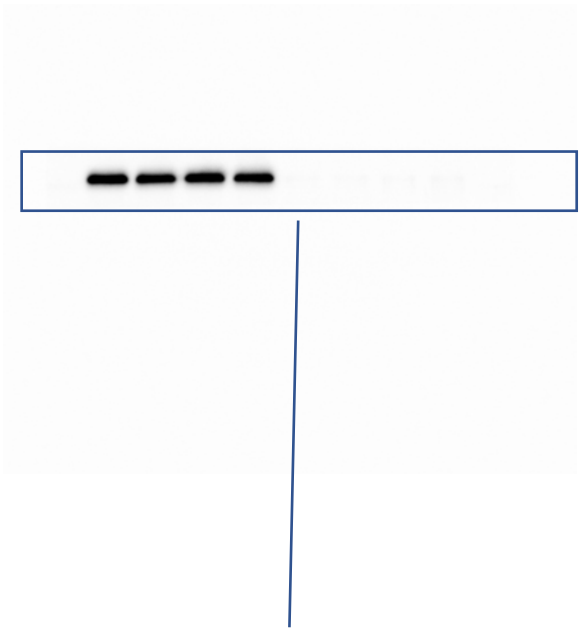

Figure 6K (The biological replicate-3.)

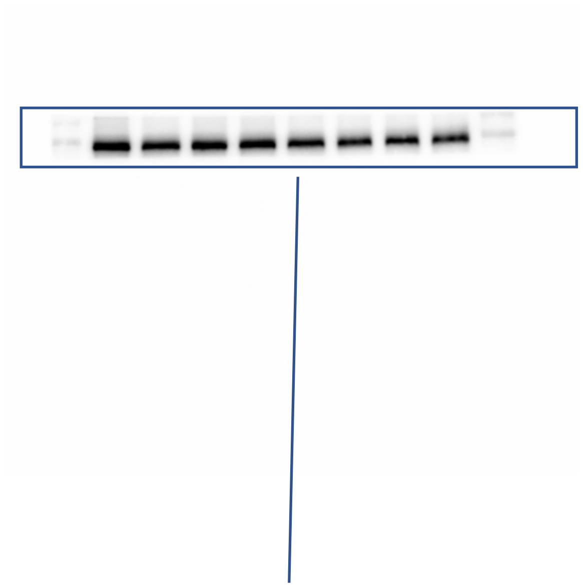

β-catenin

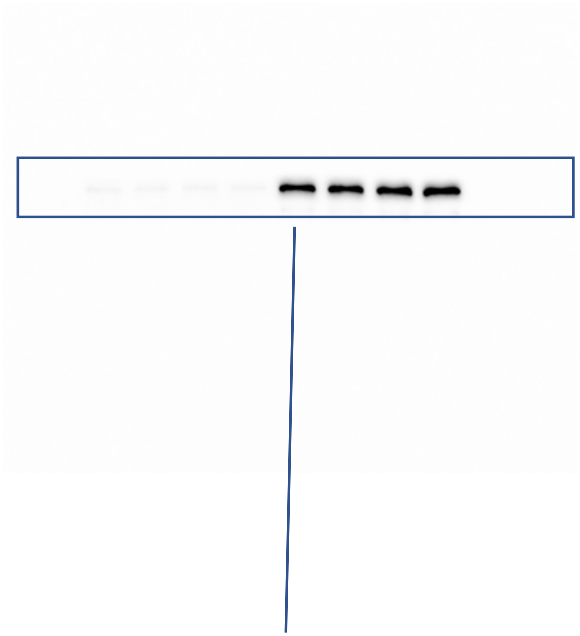

LAMINB

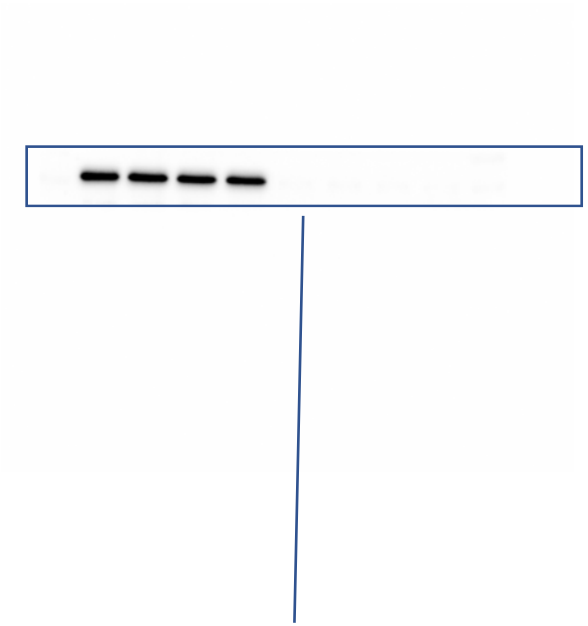

GAPDH
